# Stress analysis in a bone fracture fixed with topology-optimised plates

**DOI:** 10.1007/s10237-019-01240-3

**Published:** 2019-10-24

**Authors:** Abdulsalam Abdulaziz Al-Tamimi, Carlos Quental, Joao Folgado, Chris Peach, Paulo Bartolo

**Affiliations:** 1 Industrial Engineering Department, College of Engineering, Kind Saud University, Riyadh, Saudi Arabia; 2 School of Mechanical, Aerospace and Civil Engineering, The University of Manchester, Manchester, UK; 3 IDMEC, Instituto Superior Técnico, Universidade de Lisboa, Lisbon, Portugal; 4 Manchester University NHS Foundation Trust, Manchester, UK

**Keywords:** Fixation plates, Stress analysis, Stress shielding, Topology optimisation

## Abstract

The design of commercially available fixation plates and the materials used for their fabrication lead to the plates being stiffer than bone. Consequently, commercial plates are prone to induce bone stress shielding. In this study, three-dimensional fixation plates are designed using topology optimisation aiming to reduce the risk of bone stress shielding. Fixation plate designs were optimised by minimising the strain energy for three levels of volume reduction (i.e. 25%, 45% and 75%). To evaluate stress shielding, changes in bone stress due to the different fixation plate designs were determined on the fracture plane of an idealised shaft of a long bone under a four-point bending load considering the effect of a patient walking with crutches of a transverse fractured tibia. Topology optimisation is a viable approach to design less stiff plates with adequate mechanical strength considering high volume reductions, which consequently increased the stress transferred to the bone fracture plane minimising bone stress shielding.

**Electronic supplementary material:**

The online version of this article (10.1007/s10237-019-01240-3) contains supplementary material, which is available to authorized users.

## Introduction

Stress shielding is an important phenomenon that must be considered during design optimisation of fracture fixation plates to minimise the risk of bone resorption and plate failure (Prasad et al. 2017). It is the result of the stiffness mismatch between the most commonly used metallic fracture fixation plates and bones (e.g. Young’s modulus of Ti–6Al–4V is around 120 GPa and cortical bone is 15–25 GPa), which strongly determines the bone remodelling process whereby, according to Wolff’s law, bone adapts to the forces acting upon it (Ridzwan et al. 2007; Elias et al. 2008; Quental et al. 2014). This means that the load distribution in the bone-plate interface during healing will be uneven, mainly supported by the bone plate and screws. This will shield the bone from the stress stimulus required to provide adequate bone healing and eventually cause bone resorption and implant loosening, in a phenomenon known as “stress shielding” (Ridzwan et al. 2007; Prasad et al. 2017).

Stress shielding in bone is a common problem induced by mild- to high-load-bearing medical implants and can be reduced by redesigning the medical implant (Ramakrishna et al. 2004; Galbusera et al. 2009). The use of topology optimisation is gaining significant attention due to the ability to automatically generate optimal redesigns for a given design, considering different loading conditions and volume reduction constraints. Several authors demonstrated the feasibility of topology optimisation for the redesign of orthopaedic medical implants to minimise stress shielding such as femur hip joints (Ridzwan et al. 2006; Fraldi et al. 2010; Saravana and George 2017), spine (Chuah et al. 2010) and pelvic prostheses (Iqbal et al. 2019). In these cases, results showed improved load transfer to the bone in the case of optimised implants. Similarly, Liu et al. (2017) used topology optimisation to design mandible fixation plates with adequate biomechanical performance. The optimised designs obtained through topology optimisation present complex internal/porous structures difficult to produce using conventional manufacturing technologies (Iqbal et al. 2019). Additive technology is the ideal technique to produce these plates, not only due to the ability to produce very complex shapes, but also due to the fact that the use of additive manufacturing allows to reduce material waste, part fabrication without the use of complex tooling, being the ideal technology for mass personalisation (Parthasarathy 2015; Murr 2016). For metal fixation plates, the ideal additive manufacturing technologies are selective laser melting (SLM) and electron beam melting (EBM) (Calignano et al. 2019; Yuan et al. 2019).

A valid concern when reducing stiffness of implants is whether the change in biomechanical characteristics has a negative effect on the stability at the fracture site which might affect bone healing. In order to ensure appropriate stresses imposed on the bone, fixation plates’ stiffness should be optimised whilst maintaining plate stability during the healing process.

Therefore, this paper, as the authors’ best of knowledge, is the first study investigating the use of topology optimisation to design fixation plates that minimise stress shielding and promote load transfer to the bone fracture plane, thus stimulating bone remodelling. Two different fixation plates were considered (four- and eight-screw holes) and were optimised for different loading conditions (bending, compression, torsion and a combined load) and three volume reductions (25%, 45% and 75%). The optimised plates were evaluated using finite element analyses considering a tibia-like bone shape model under a bending loading condition to study the induced stresses on the defined bone fracture plane.

## Modelling and simulation

### Optimisation and mechanical evaluation

**Fig. 1:**
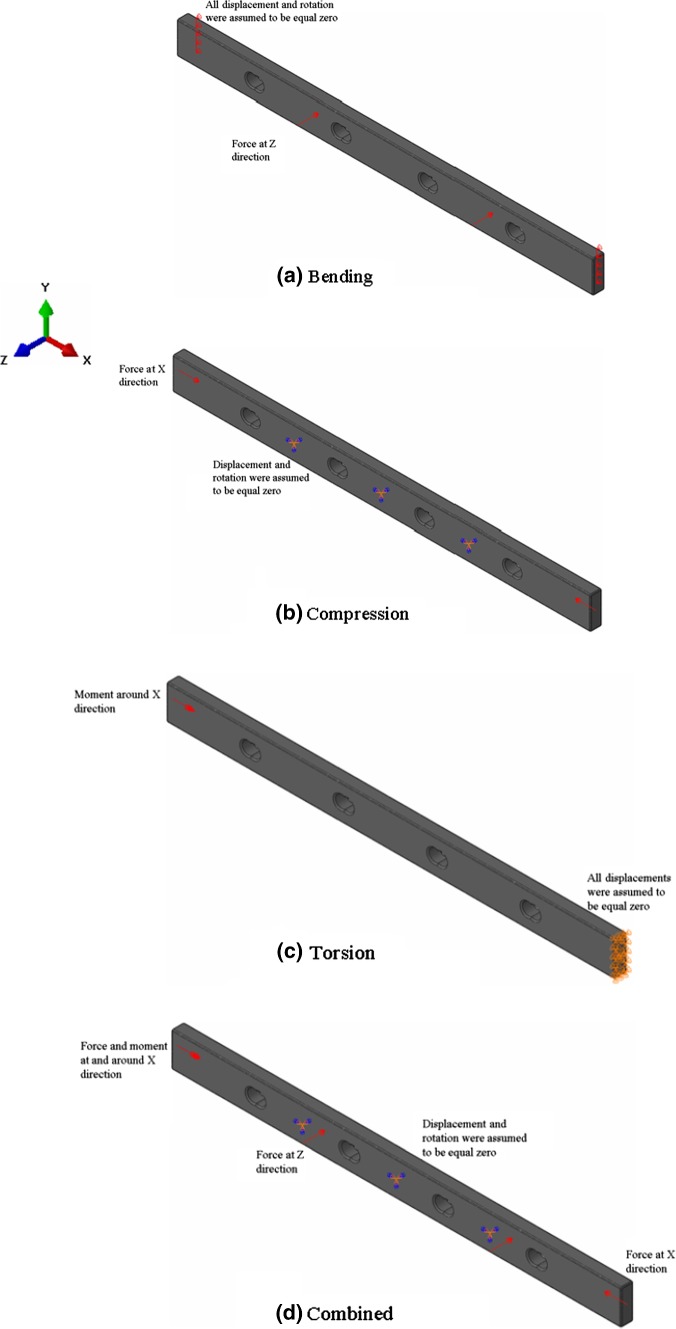
Load and boundary conditions considered for the fixation plate optimisation of **a** four-point bending load, **b** uniaxial compression, **c** torsional and **d** combination of the bending, compression and torsion loads

The Solid Isotropic Microstructure with Penalisation (SIMP) method was applied to redesign two different fixation plates with four- and eight-screw holes. Initial designs were created in Solidworks (Dassault Systèmes, Waltham, MA, USA) considering generic locking compression fixation systems for treating long bones for midshaft fractures with a length of 180 mm, width of 14 mm and thickness of 5 mm. Three volume reductions (25%, 45% and 75%), and different loading conditions (bending, compression, torsion and a combination of all these loads), were considered as shown in Fig. 1. A frozen region was considered on the screw hole region to keep their shape. Simulations were performed considering quadratic hexahedral meshes with around 50,000 elements.

Mathematically, the SIMP formulation can be described as follows (Bendsøe and Sigmund, 2004):

$$ \mathop {\hbox{min} }\limits_{{\rho_{e} }} C\left( {\rho_{e} } \right) = {\mathbf{f}}^{\text{T}} \cdot {\mathbf{u}} $$

1 $$ {\text{subject}}\;{\text{ to}}\;\left\{ {\begin{array}{*{20}l} {\mathop \sum \limits_{e = 1}^{N} \rho_{e} v_{e} \le V^{*} ,} \hfill \\ {\left( {\mathop \sum \limits_{e = 1}^{N} \rho_{e}^{p} {\mathbf{K}}_{e} } \right){\mathbf{u}} = {\mathbf{f}}, } \hfill \\ {0 < \rho_{0} \le \rho_{e} \le 1, } \hfill \\ \end{array} } \right. $$

where *C* is the compliance, *p* is a penalisation factor (*p* = 3), **u** is the displacement vector, **f** is the force vector, *ρ*_*e*_ is the element density, *ρ*_o_ is the initial density, V^*^ is the volume fraction, *v*_*e*_ is the volume of each element, and **K**_*e*_ is the element stiffness matrix. The topology optimisation problem was run using the TOSCA module in Abaqus (Dassault Systèmes, Waltham, MA, USA).

The mechanical behaviour of both initial and optimised designs was investigated through finite element analyses, assuming elastic behaviour and homogeneous and isotropic plates. Numerical simulation was used to determine the equivalent stiffness in a four-point bending setting according to the British standards (BS 3531-23.1:1991 ISO 9585:1990). In this case, the equivalent bending stiffness is determined according to the following equation:

2 $$ E_{\text{B}} = \frac{{\left( {4h^{2} + 12ha + a^{2} } \right) \cdot K \cdot h}}{24}\;({\text{N}}\,{\text{m}}^{2} ) $$

where *h* is the distance between the force points, *a* is the span between the force and support points, and *K* is the stiffness calculated as follows:

3 $$ K = \frac{\text{RF}}{D}\;({\text{N/m}}) $$

where RF is the average reaction force in the *z* axis (along with the fixation plate thickness) at the constraint points and *D* is the maximum displacement.

Changes in the equivalent stiffness between the optimised fixation plates and the initial designs were calculated using the following equation:

4 $$ \begin{aligned} &\Delta {\text{equivalent stiffness}}\,( \%) \\ &= \frac{{\left( {{\text{Plate stiffness}}^{\text{Optimised}} - {\text{Plate stiffness}}^{\text{Initial}} } \right)}}{{{\text{Plate stiffness}}^{\text{Initial}} }} \end{aligned} $$

### Stress analysis of a bone model

In order to determine the stresses in the fracture plane of a bone, which provides an indication of the stress shielding effect of the plates, a bone-plate construct (i.e. the assembly of the fracture plate to the bone with screws) was considered. For simplicity, no fracture gap was imposed, and the plate was assumed to fixate a transverse fractured tibia bone. Only the cortical bone region was considered, and a hollow cylinder region with an external diameter of 24 mm and an internal diameter of 12 mm was assumed for simulation purposes.

**Fig. 2:**
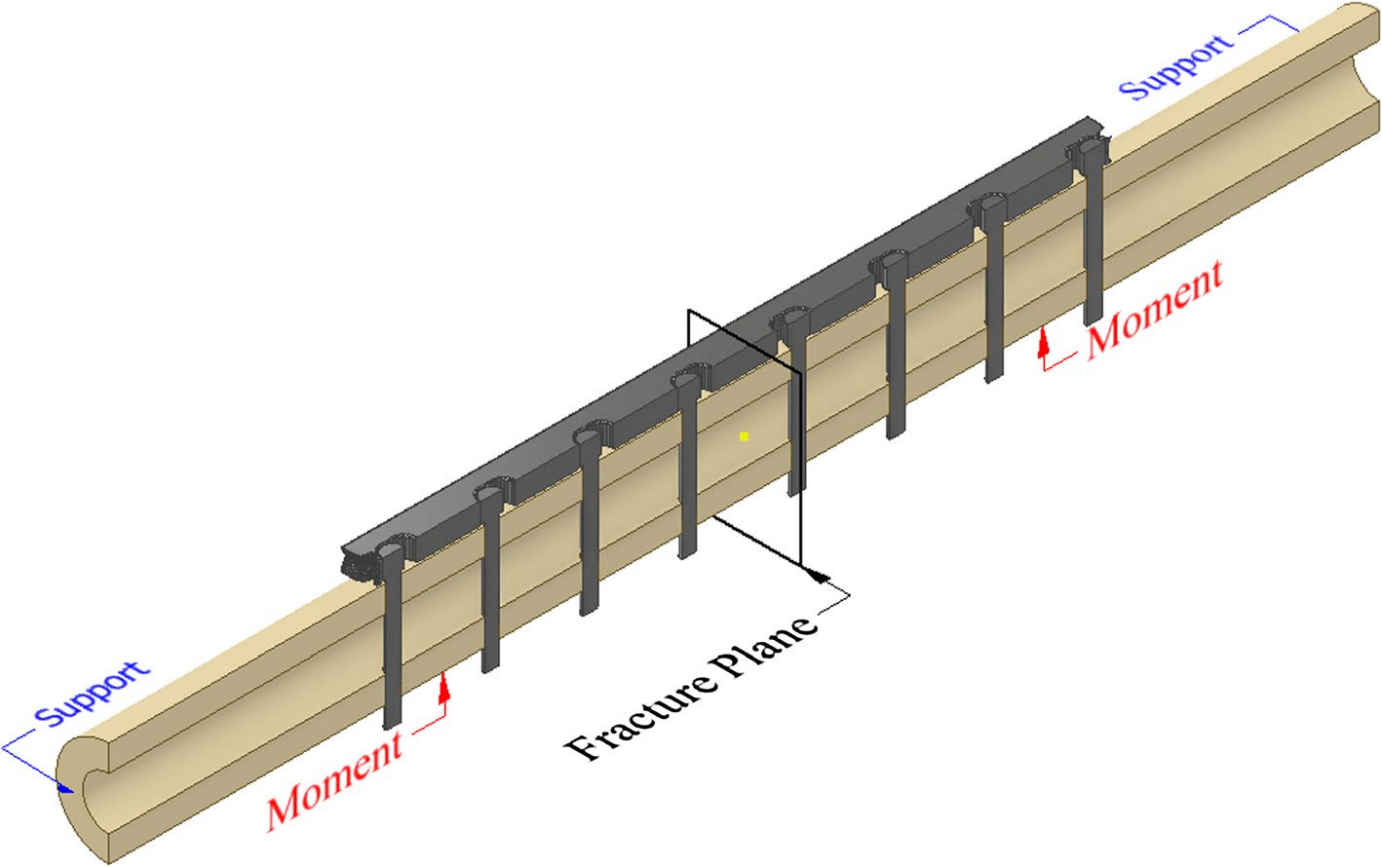
Bone-plate construct considered to determine stresses at the fracture plane

All 3D geometric parts, i.e. cortical bone, screws and initial fixation plates, were modelled in Solidworks (Dassault Systèmes, Waltham, MA, USA). Each screw has a 5-mm-diameter head and a main body with 3.5 mm of diameter and 34 mm of total length. Both fracture plates and screws were assumed to be made of Ti–6Al–4V. For the cortical bone, a Young’s modulus of 18 GPa and a Poisson’s ratio of 0.3 were assumed (Santos et al. 2018). In order to avoid high computational costs, only half of the bone-plate construct was considered, as illustrated in Fig. 2. To simulate the Locking Compression Plate technique, the finite element model considered the bone-plate construct with a gap of 0.5 mm between the bone and plate (i.e. no contact). The screw heads were securely locked to the plate and the screws tied to the bone. Quadratic hexahedral elements were considered for the bone model region of interest (i.e. fracture plane) and quadratic tetrahedron elements for the plates, screws and the bone region outside of the fracture plane. Two equally distributed moments of 20 Nm were applied along the horizontal axis of the bone, simulating the moment load happening on the tibia during the swing phase (i.e. 10% of the body weight) in patients walking with crutches (Ramakrishna et al. 2004; Wehner et al. 2009; Kim et al. 2011). To prevent rigid body motion, the extremity faces of the bone were fully constrained.

In addition, since the mechanical strength of the topology-optimised fixation plates is important to assess their stability during healing, a mechanical strength analysis was performed based on the materials yield strength, considering the yield strength of the Ti–6Al–4V as ~ 860 MPa (Elias et al. 2008). The von Mises stresses on the topology-optimised fixation plates were used to determine their mechanical strength and, consequently, to investigate their stability.

## Results

The changes in the equivalent stiffness between the optimised and initial designs are shown in Table 1. Optimised designs obtained through topology optimisation are presented as supplementary material (Figs. S1 and S2). The equivalent bending stiffness change increases as the volume reduction increases. For the same volume reduction and plates with different number of holes, there is no clear trend in terms of the equivalent stiffness change. Also, as expected, plates optimised considering bending loading conditions exhibit the least reduced equivalent stiffness change. The highest decreases were observed for the 75% volume reduction with combined load for the four-hole and eight-hole.

**Table 1:** Change in the equivalent bending stiffness in comparison with the initial values for four- and eight-hole plates (19.27 and 16.22 N m^2^, respectively)

Plate	Volume reduction (%)	Hole numbers	
		Equivalent stiffness change (%)	
		Four-hole plate	Eight-hole plate
Bending	25	− 3	− 2
	45	− 5	− 5
	75	− 20	− 61
Compression	25	− 15	− 15
	45	− 17	− 28
	75	− 49	− 71
Torsion	25	− 3	− 31
	45	− 9	− 33
	75	− 49	− 64
Combined	25	− 4	− 4
	45	− 57	− 7
	75	− 92	− 87

**Fig. 3:**
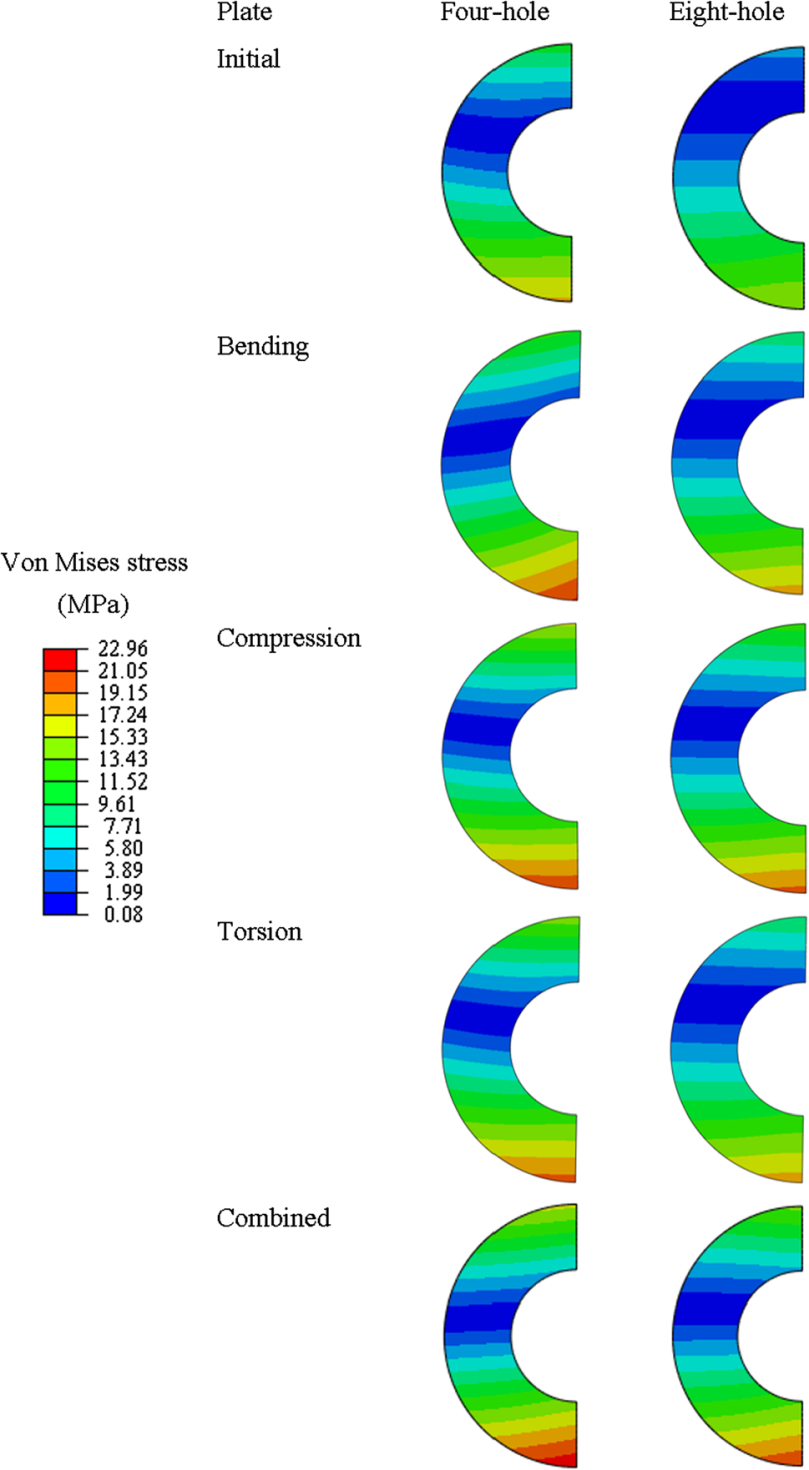
von Mises stresses at the bone fracture plane resulted from the initial designs and all of the 75% volume reduction optimised plates

**Fig. 4:**
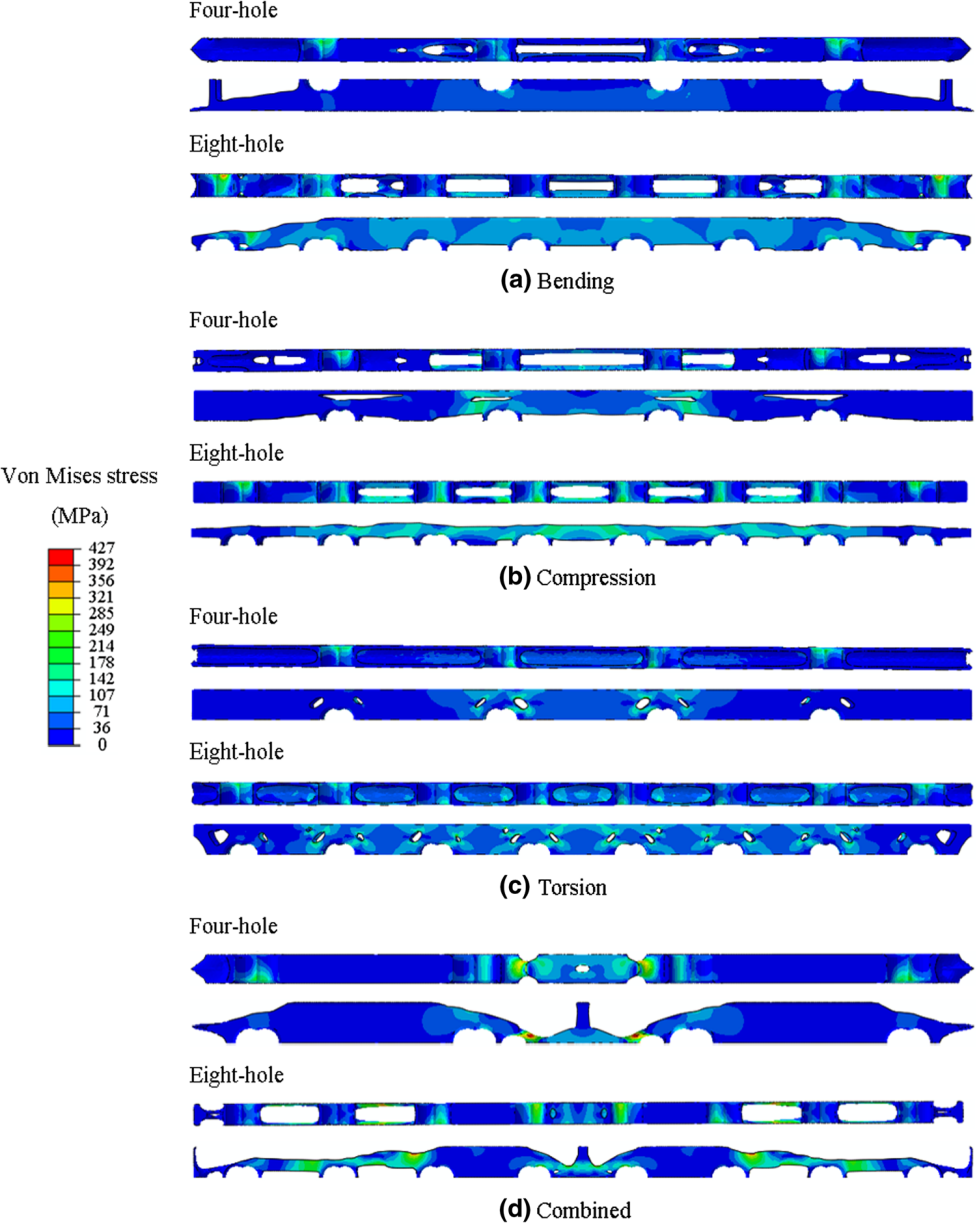
Stress distribution on the optimised four- and eight-screw hole plates with 75% volume reduction and different loading conditions **a** bending, **b** compression, **c** torsion and **d** a combined load

The maximum von Mises stresses from the bone-plate construct at the fracture plane, considering both the initial and topology-optimised fixation plates, are presented in Table 2. The stress distribution and magnitude at the bone fracture plane are presented in Fig. 3, for the initial designs and only for the 75% volume reduction optimised plates, which is the case causing the maximum von Mises stresses on the bone at the fracture plane. Overall, the stresses in the bone increase when topology-optimised plates are used. The most substantial increase in stresses at the fracture plane was observed for the combined loading conditions and 75% volume reduction plates. In comparison with the initial designs, the maximum stresses at the fracture plane increased by 31% for the four-hole plate and 37% for the eight-hole plate in the combined case with a 75% of volume reduction. The stress distribution shows that less stiff plates produce higher compressive stresses (in the plate-bone interface due to the bending load) at the fracture plane. Moreover, the neutral axis with the less stiff plates becomes closer to that of the bone’s when compared with high stiff plates. In terms of plate mechanical stability (i.e. mechanical strength), the stresses among the least stiff fixation plates (75% of volume reduction) are shown in Fig. 4. The maximum stresses occur on both four-hole and eight-hole designs with a combined load and 75% of volume reduction. Minimum values of the von Mises stresses for all plates occur for compression loads and 75% of volume reduction.

**Table 2:** Maximum von Mises stresses on the bone at the fracture plane for all considered designs

Plate	Volume reduction (%)	Hole numbers	
		von Mises stress (MPa)	
		Four-hole plate	Eight-hole plate
Initial designs	N/A	17.59	15.37
Bending	25	17.95	15.38
	45	19.93	15.93
	75	21.41	18.15
Compression	25	17.94	15.46
	45	19.89	15.58
	75	20.79	19.91
Torsion	25	17.77	15.73
	45	19.92	15.81
	75	20.03	18.25
Combined	25	17.61	15.40
	45	17.88	15.75
	75	22.96	21.07

## Discussion and conclusions

Results show that through topology optimisation it is possible to design less stiff fixation plates, resulting in higher loads being transferred to the bone fracture plane, whilst maintaining the plate’s ability to withstand stresses. By considering a maximum of 75% of volume reduction for plates containing different screw holes, it is possible to increase the load transfer to up to 37% in comparison with the initial plates. This reduces stress shielding, and likely bone loss, to promoting secondary healing, that promotes callus formation and bone formation (Woo et al. 1977; Goodship and Kenwright 1985; Claes et al. 1997).

Maximum von Mises stresses were observed in plates optimised for combined loading conditions and 75% of volume reduction. This can be explained by stress concentrations induced by the plate design presenting thin features. However, as observed, the highest stresses are still 50% lower than the yield strength of the material, guaranteeing the plate mechanical stability.

For the four-hole plates, the best performance was observed for plates with 75% of volume reduction and optimised for bending loading conditions, which enable 22% of load transfer to the bone, presenting also low von Mises stresses (221 MPa). For the eight-hole plates, a maximum load transfer of 29% and 240 MPa of von Mises stresses were observed for compression load optimised plates.

As shown, topology optimisation permits the design of less stiff and lightweight fixation plates, reducing the stress shielding effect, promoting load transfer to the bone and thus contributing to bone remodelling. However, further analysis is still required, considering for example a fracture gap and measuring the gap strains to correlate the resulted strains (i.e. relative or absolute stability) with the healing process (i.e. secondary or primary healing). Furthermore, screw threads were not considered in the simulation and their role on load transfer must be also considered. Although no critical failure was observed in the optimised designs, thin and sharp features were yet observed. Further post-processing design steps must be considered to address these features. Additional constraints must also be considered to allow further fabrication (e.g. thickness of internal features which might be difficult to produce using additive manufacturing).

## Electronic supplementary material

Below is the link to the electronic supplementary material.

Supplementary material 1 (DOCX 702 kb)
