# Supplementary material for: Stress analysis in a bone fracture fixed with topology-optimised plates
Source: Biomech Model Mechanobiol. 2019 Oct 24;19(2):693–9. doi: 10.1007/s10237-019-01240-3 (PMC7105442; doi:10.1007/s10237-019-01240-3)
Supplement: Supplementary file 1 — Supplementary material 1 (DOCX 702 kb) [file 10237_2019_1240_MOESM1_ESM.docx]

**Stress analysis in a bone fracture fixed with topology optimised plates**

Abdulsalam Abdulaziz Al-Tamimi^ab^, Carlos Quental^c^, Joao Folgado^c^ Chris Peach^bd^, Paulo Bartolo^b*^

^a^ Industrial Engineering Department, College of Engineering, Kind Saud University, Riyadh, Saudi Arabia

^b^ School of Mechanical, Aerospace and Civil Engineering, The University of Manchester, Manchester, UK

^c^ IDMEC, Instituto Superior Técnico, Universidade de Lisboa, Lisbon, Portugal

^d^ Manchester University NHS Foundation Trust, Manchester, UK

*Corresponding author: paulojorge.dasilvabartolo@manchester.ac.uk

List of emails in sequential:

Abdulsalam.altamimi@postgrad.manchester.ac.uk; carlos.quental@tecnico.ulisboa.pt;

jfolgado@tecnico.ulisboa.pt; chris@shouldersandelbows.co.uk;

paulojorge.dasilvabartolo@manchester.ac.uk

Topology optimization results obtained for the four- (Fig. S1) and eight- (Fig. S2) screw hole fracture plates under bending, compression, torsion and combined loads.

| 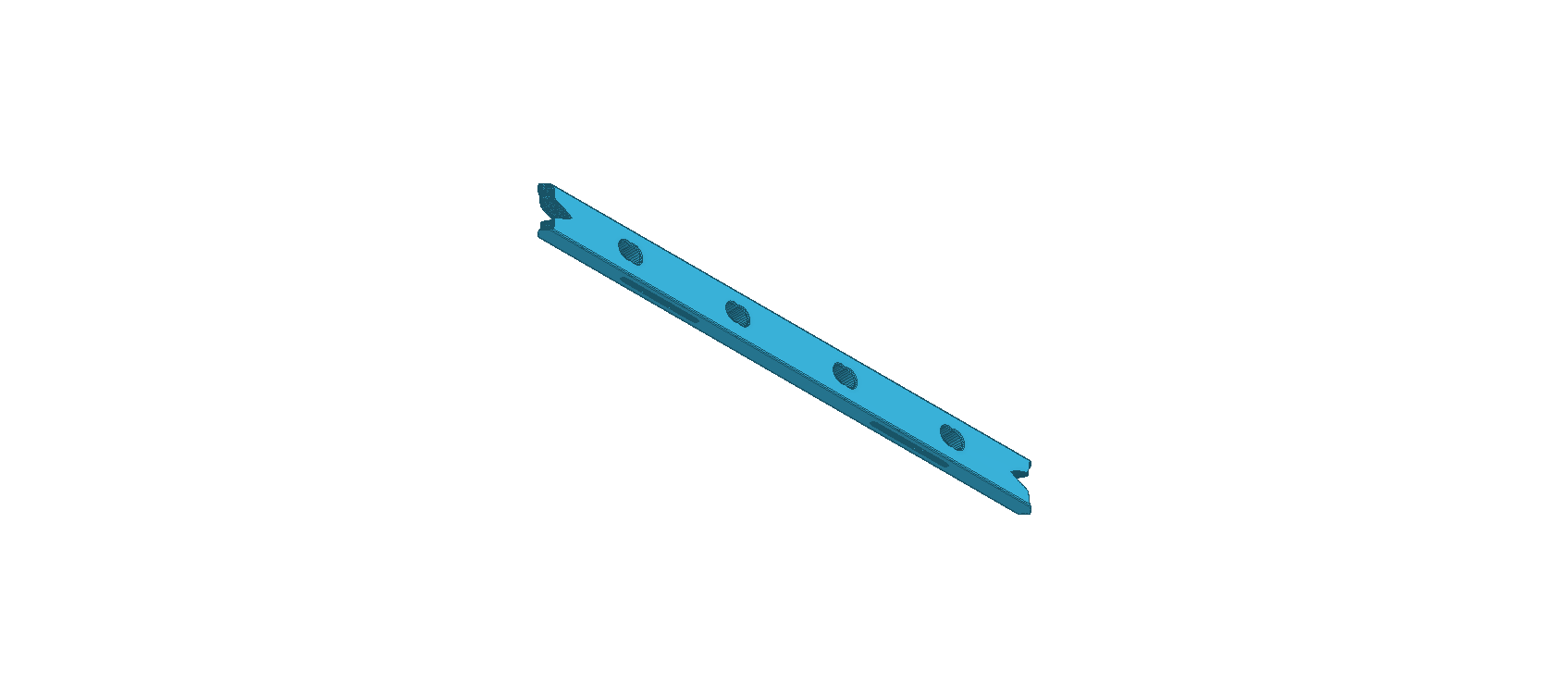  (a) | 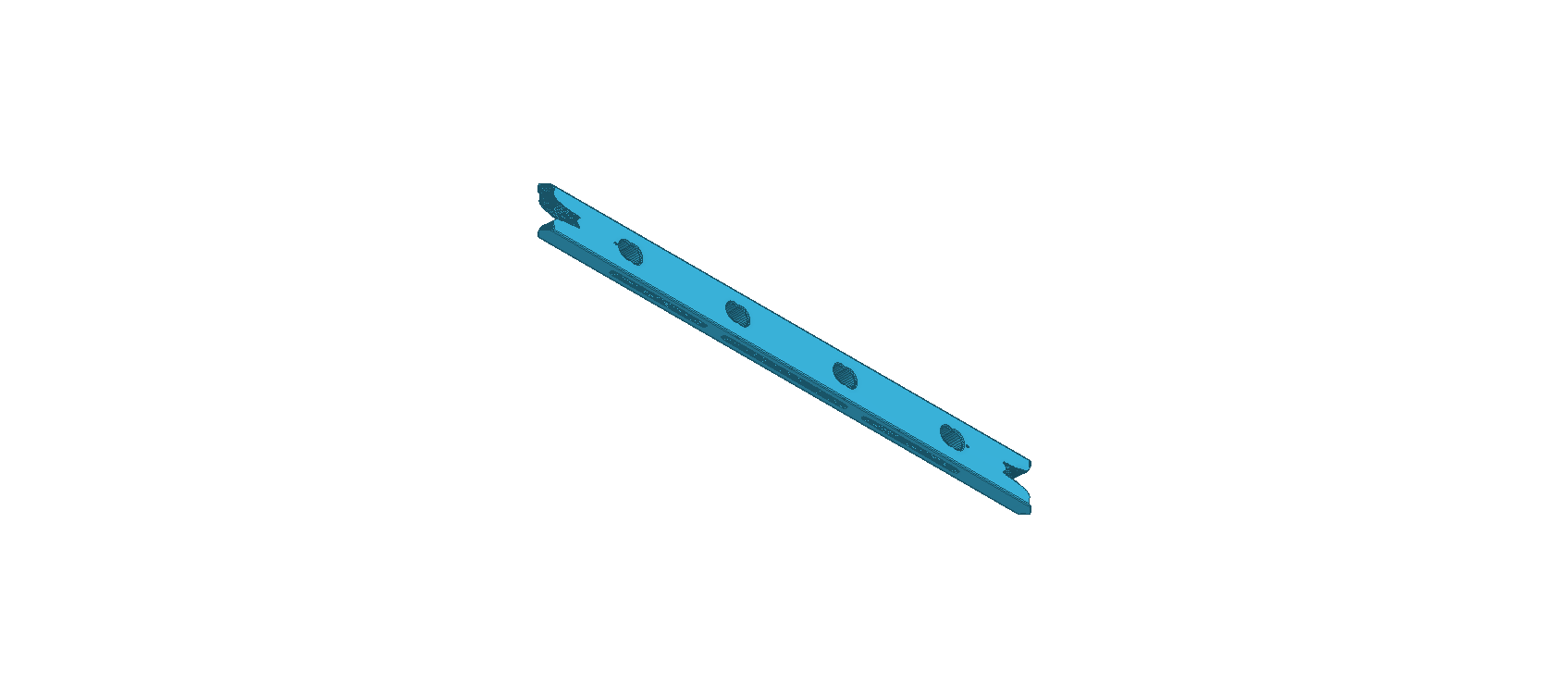  (b) | 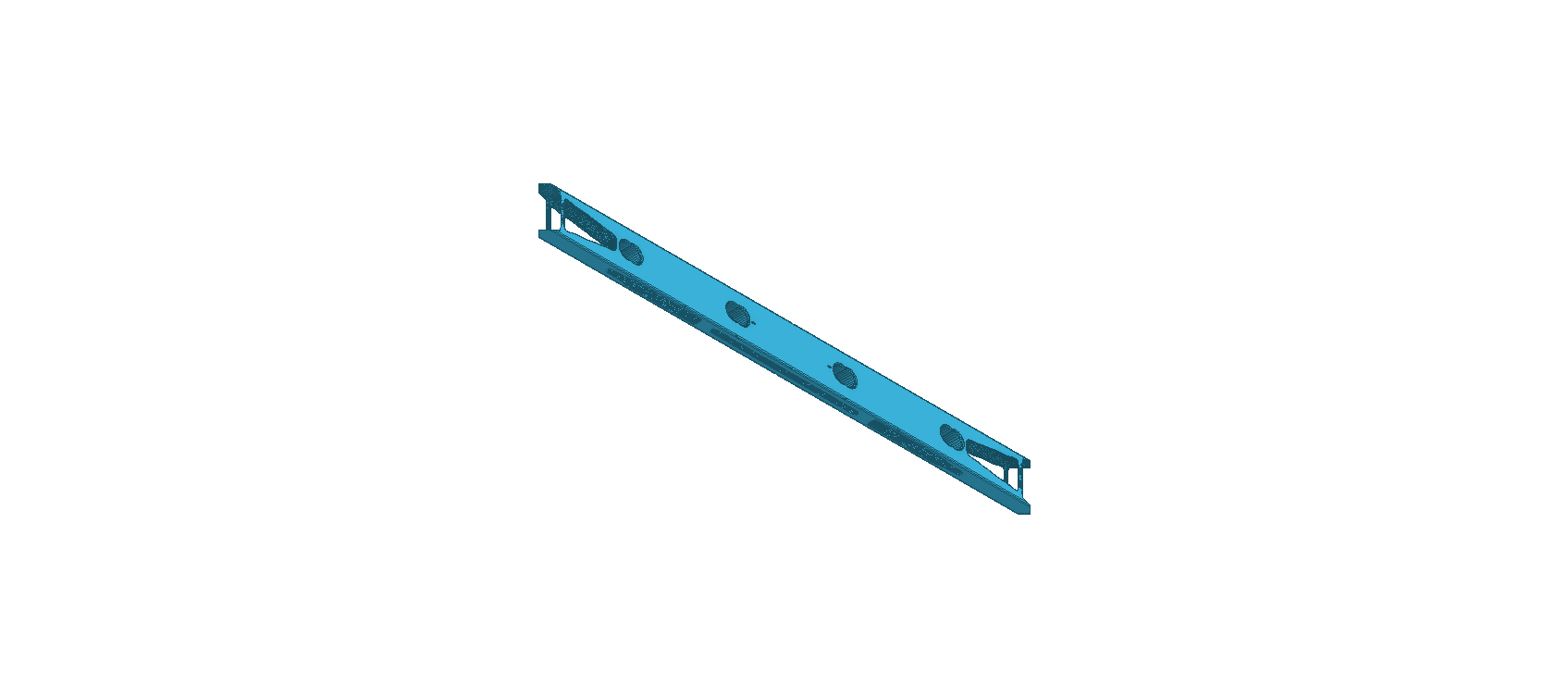  (c) |
| --- | --- | --- |
| 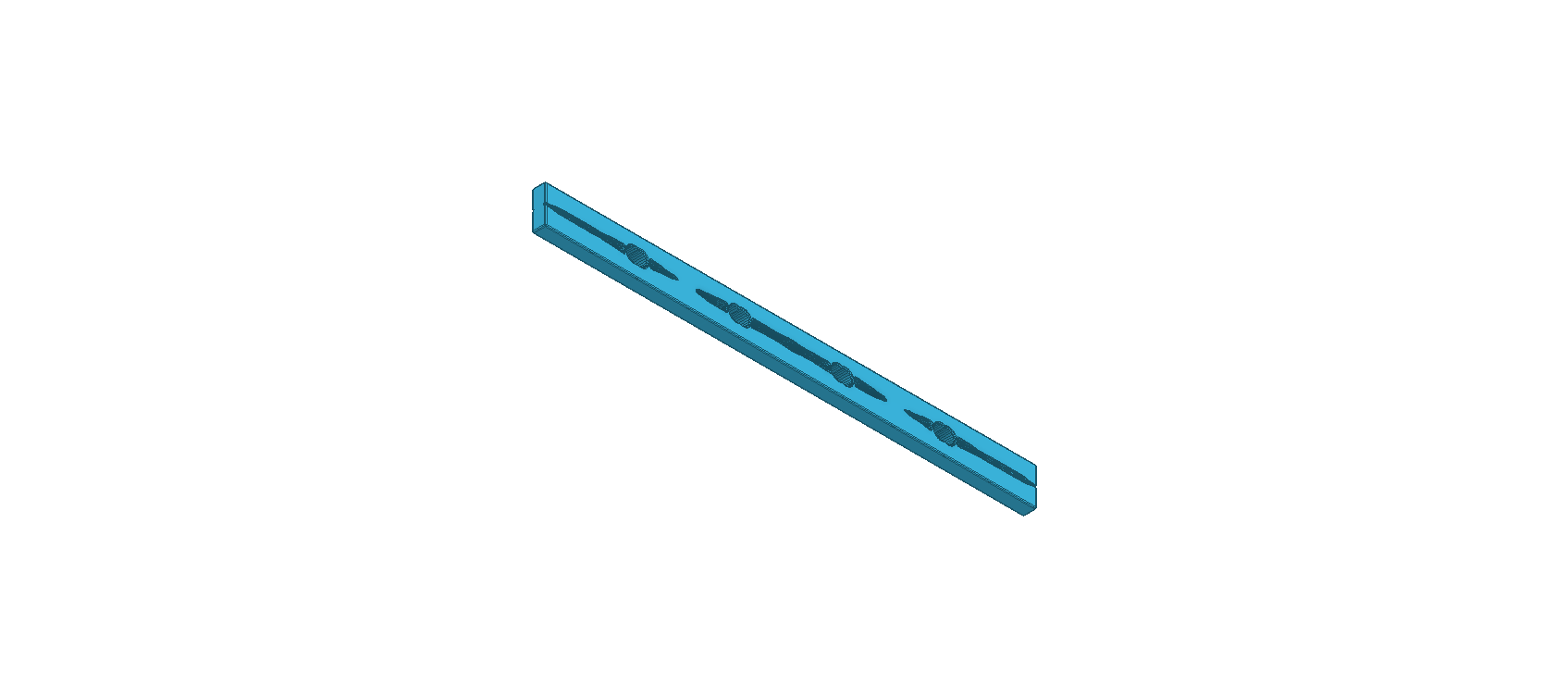  (d) | 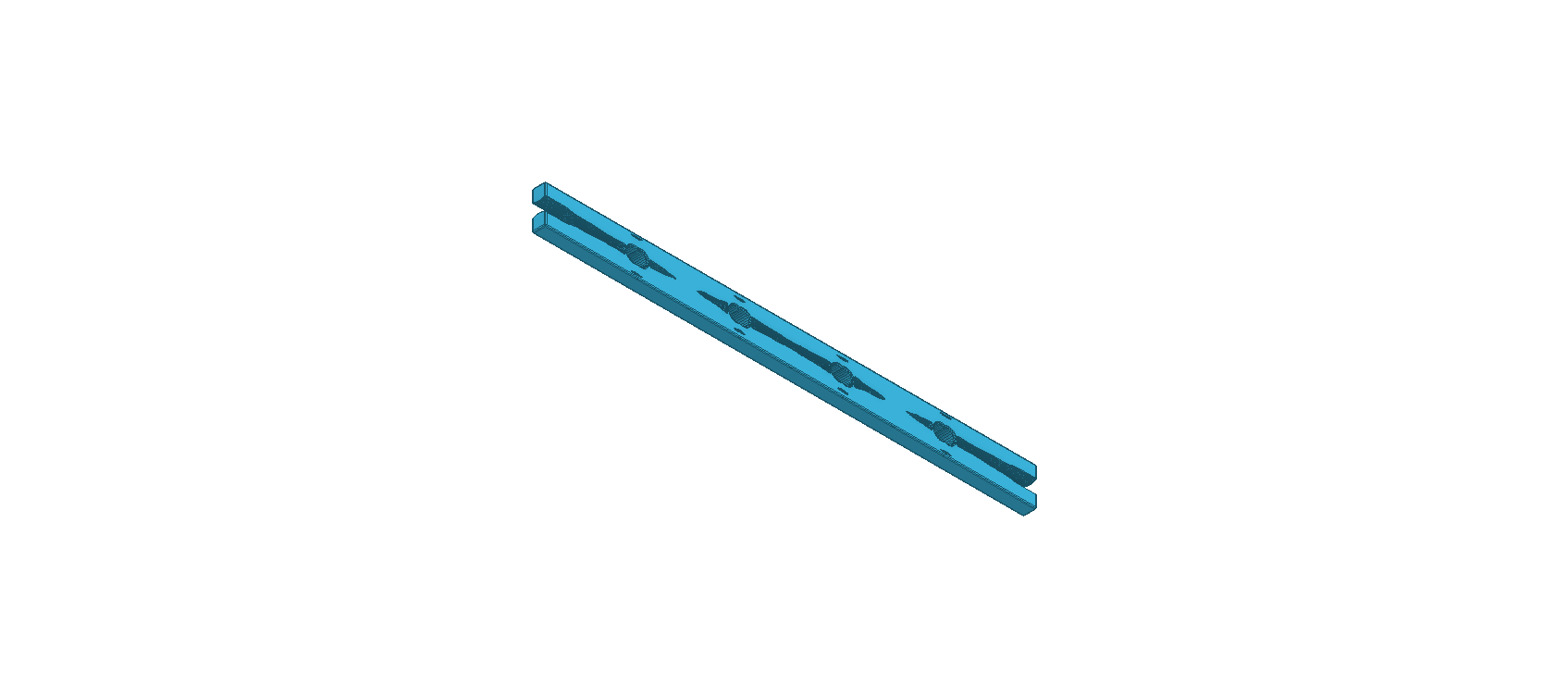 (e) | 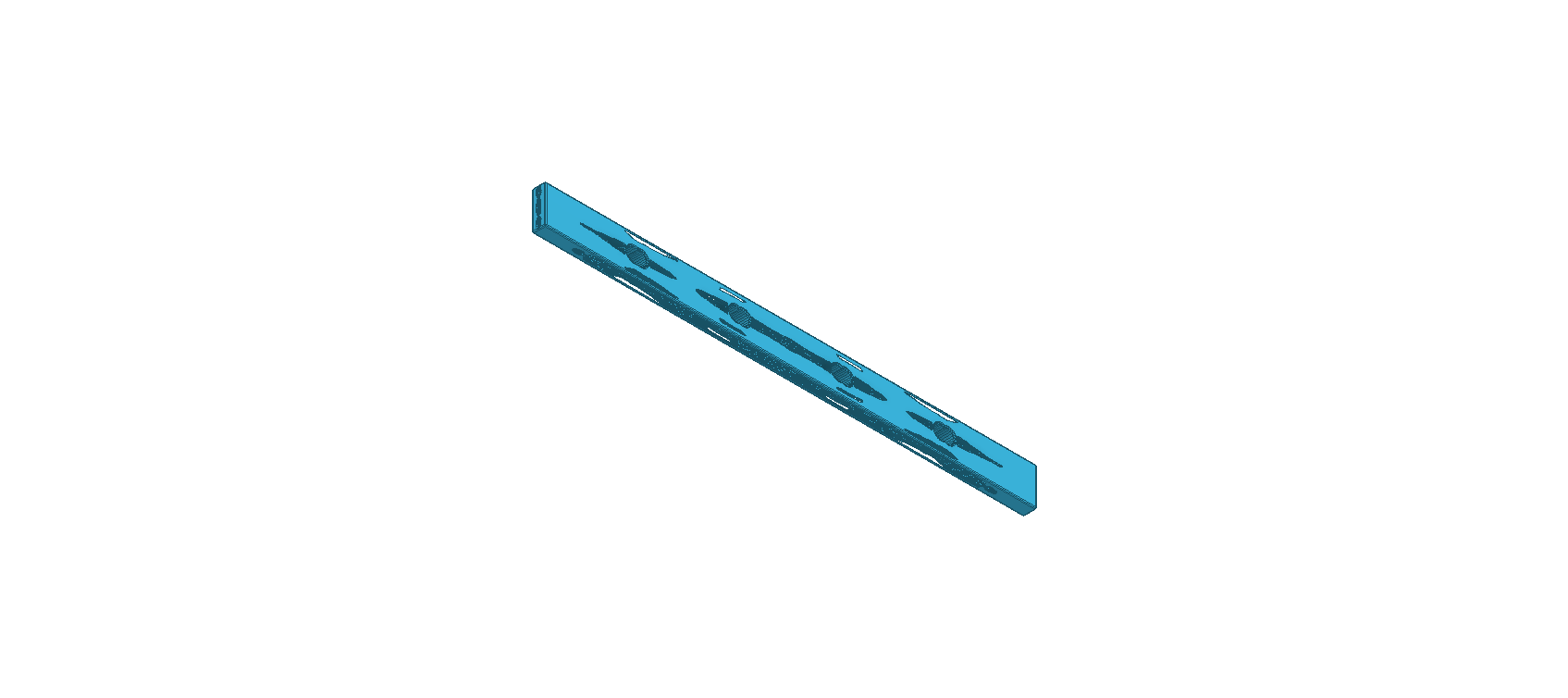  (f) |
| 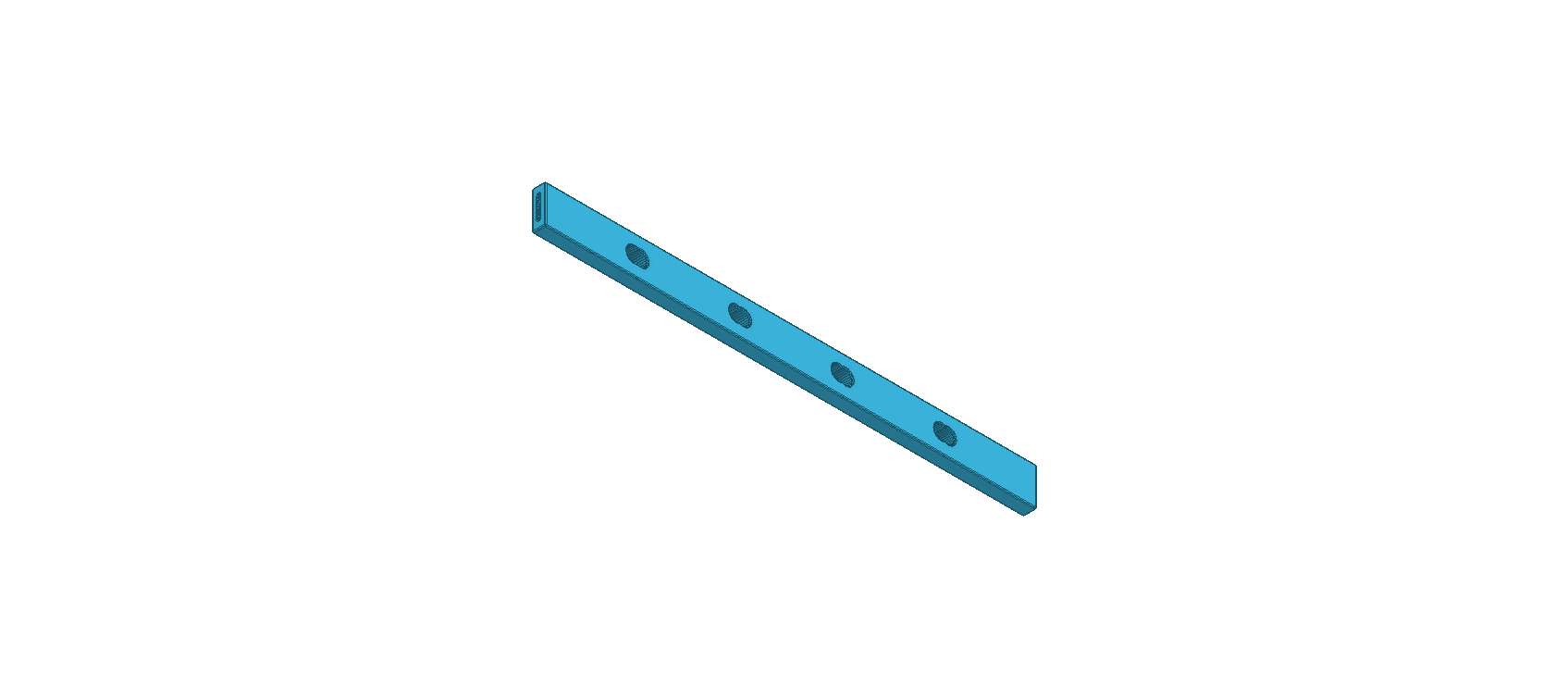 (g) | 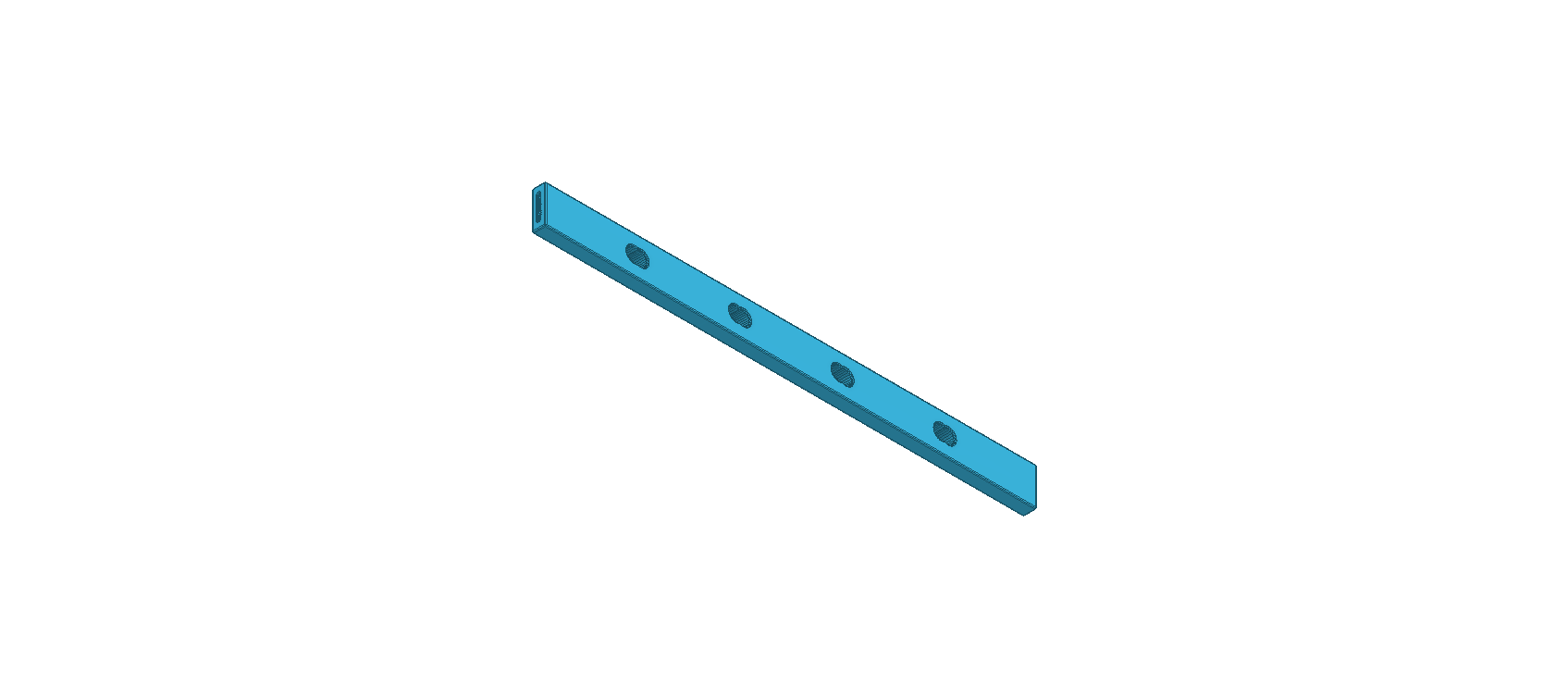  (h) | 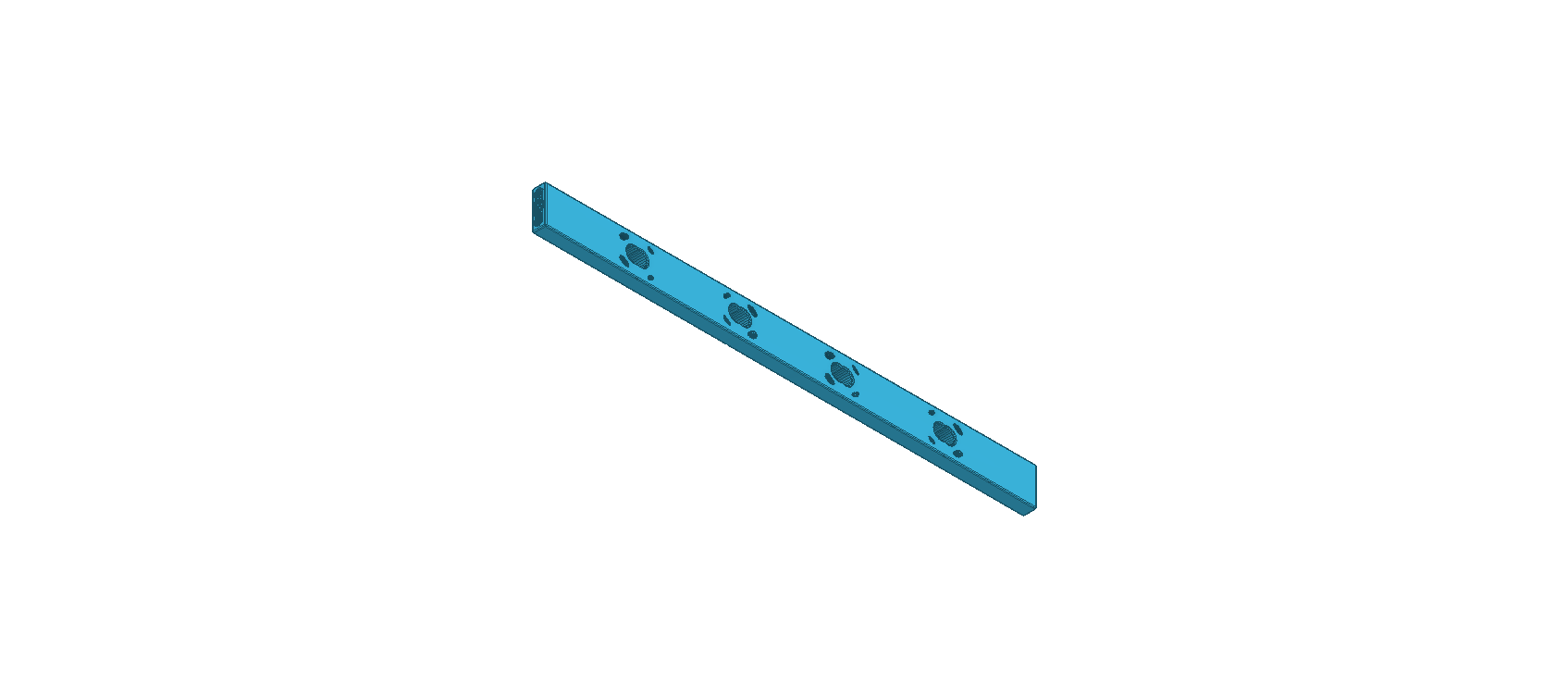  (i) |
| 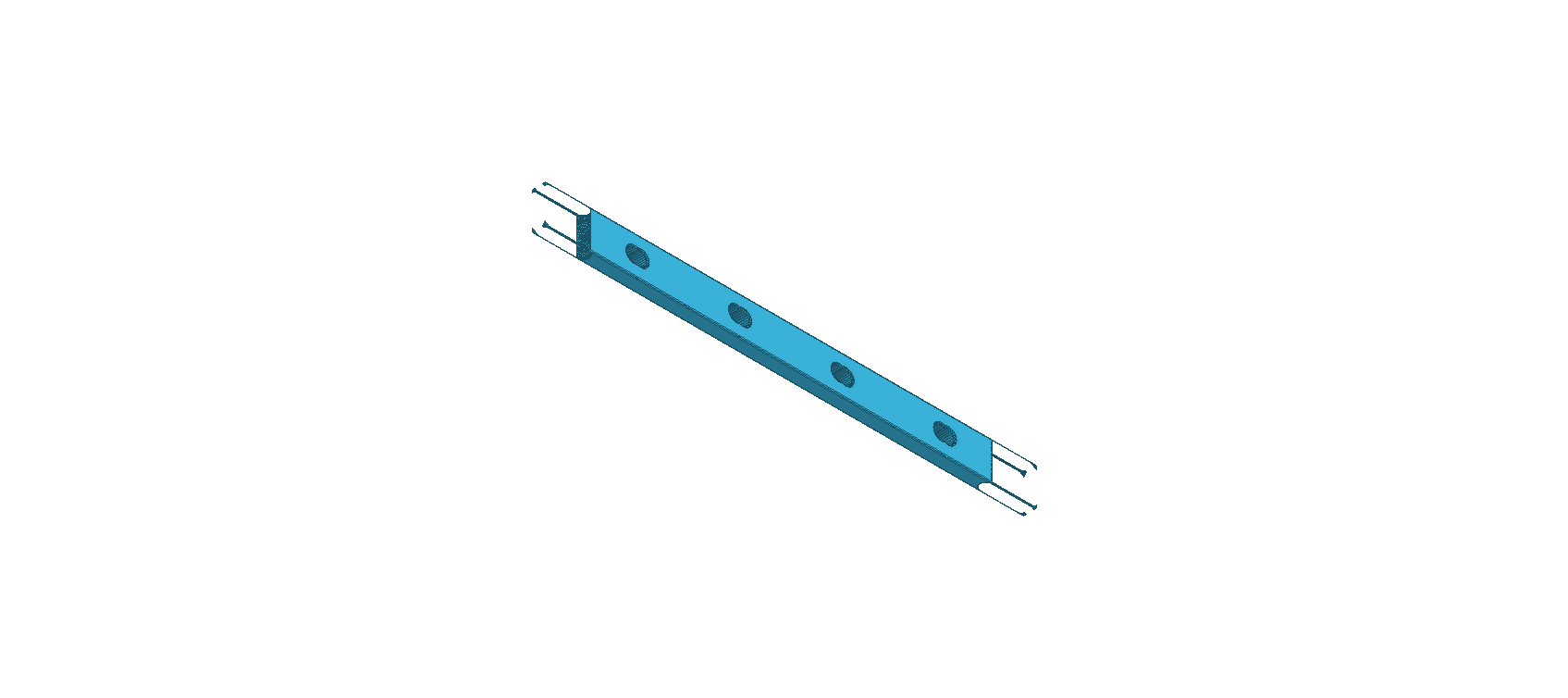 (j) | 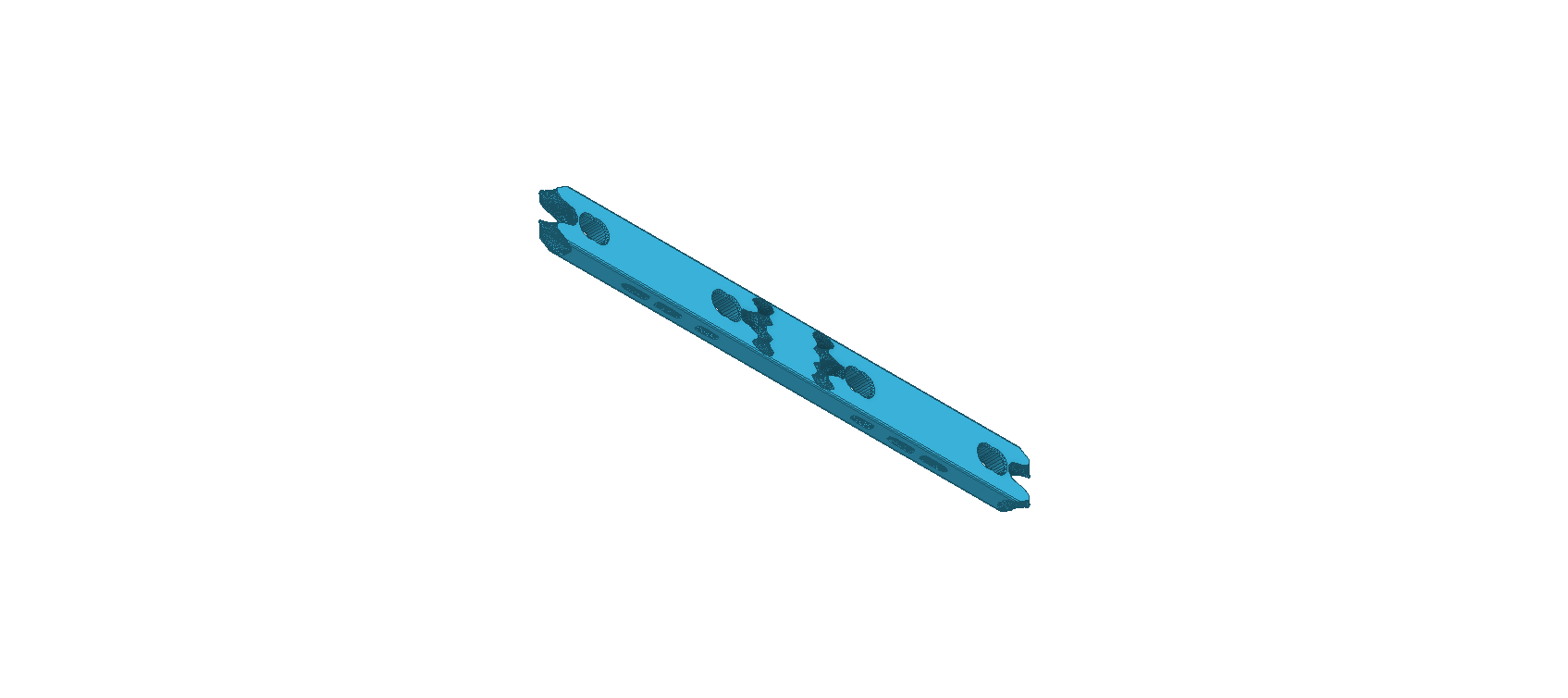  (k) | 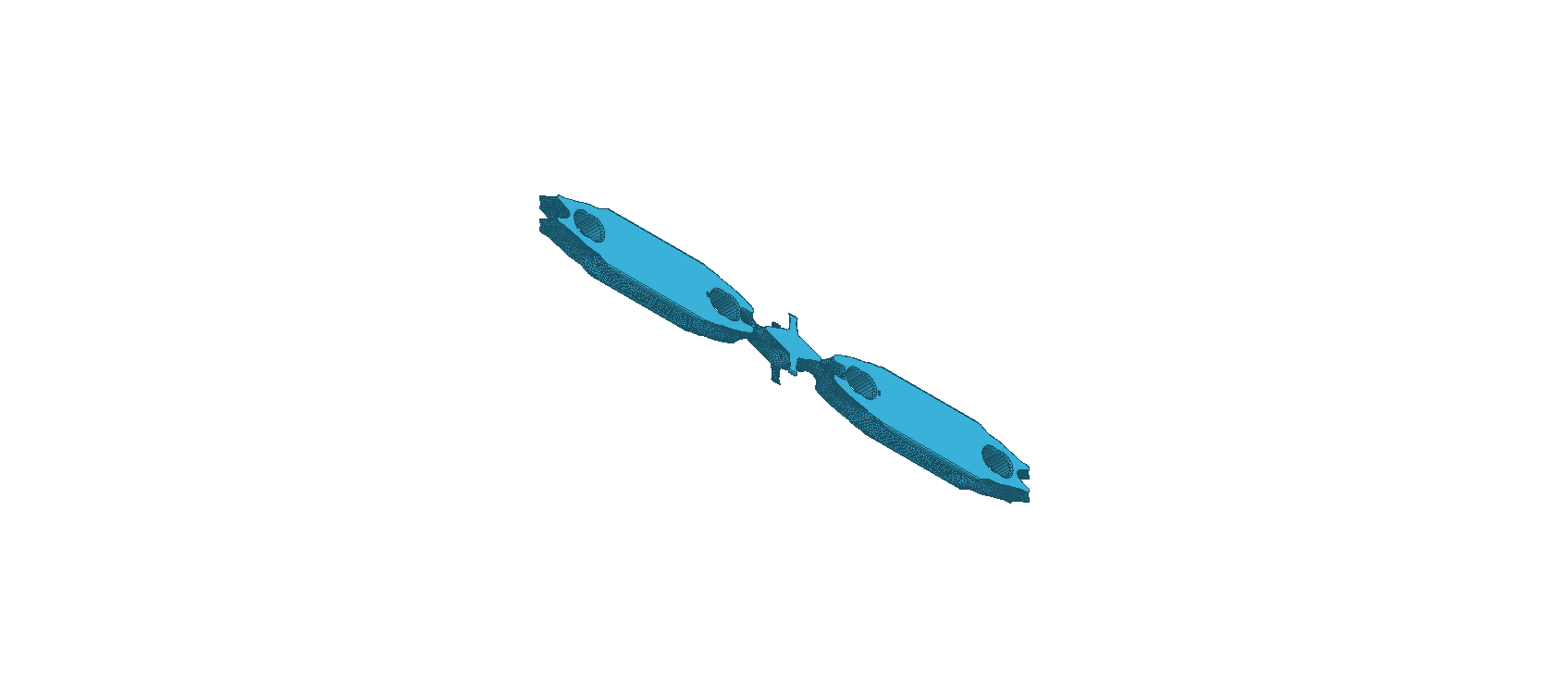  (l) |

Fig. S1 Topology optimization results of four-hole fracture plate. Under bending load: (a) 25% of volume reduction, (b) 45% of volume reduction and (c) 75% of volume reduction. Under compression load: (d) 25% volume reduction, (e) 45% of volume reduction and (f) 75% volume reduction. Under torsion load: (g) 25% of volume reduction, (h) 45% of volume reduction and (i) 75% of volume reduction. Under combined load: (j) 25% of volume reduction, (k) 45% of volume reduction and (l) 75% of volume reduction.

| 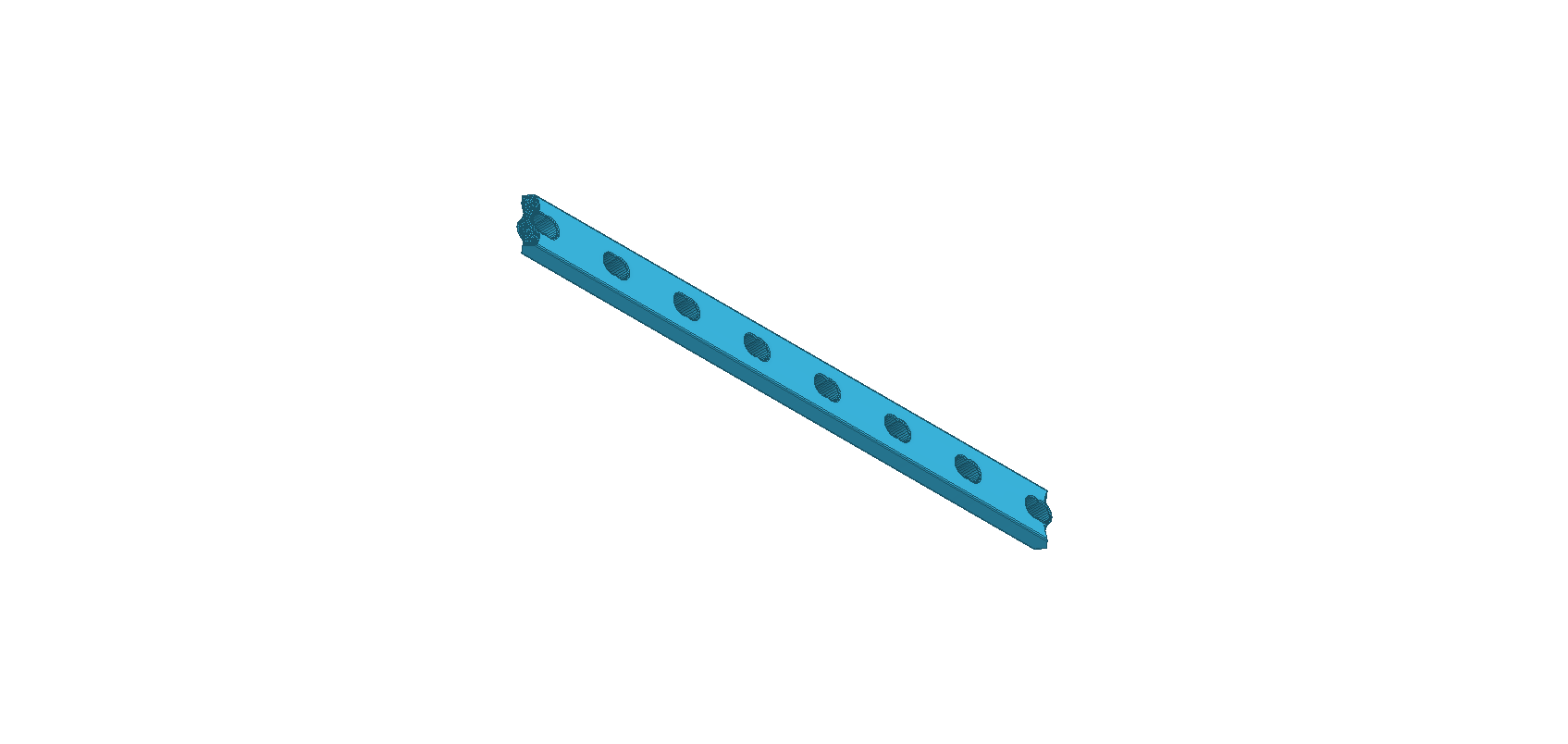  (a) | 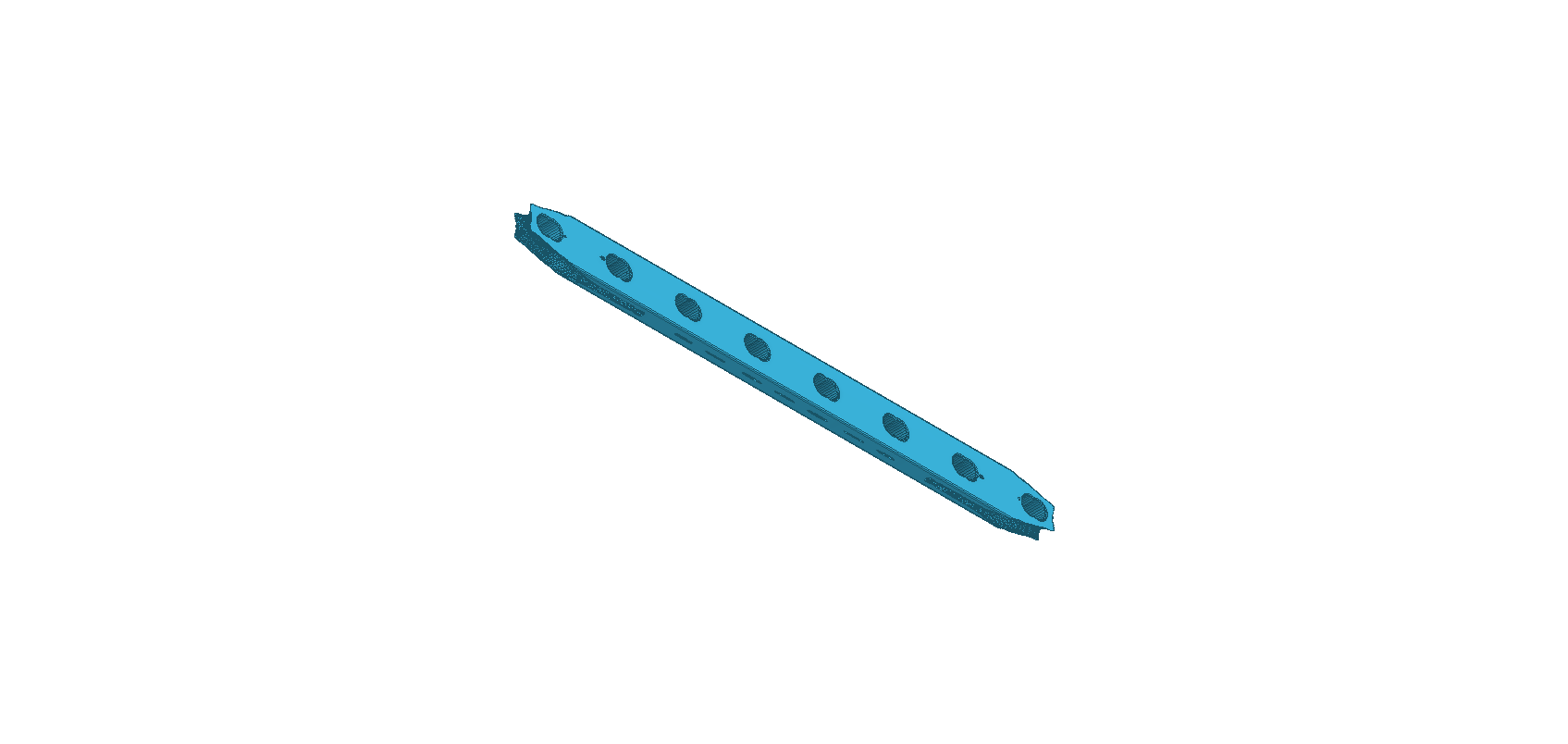  (b) | 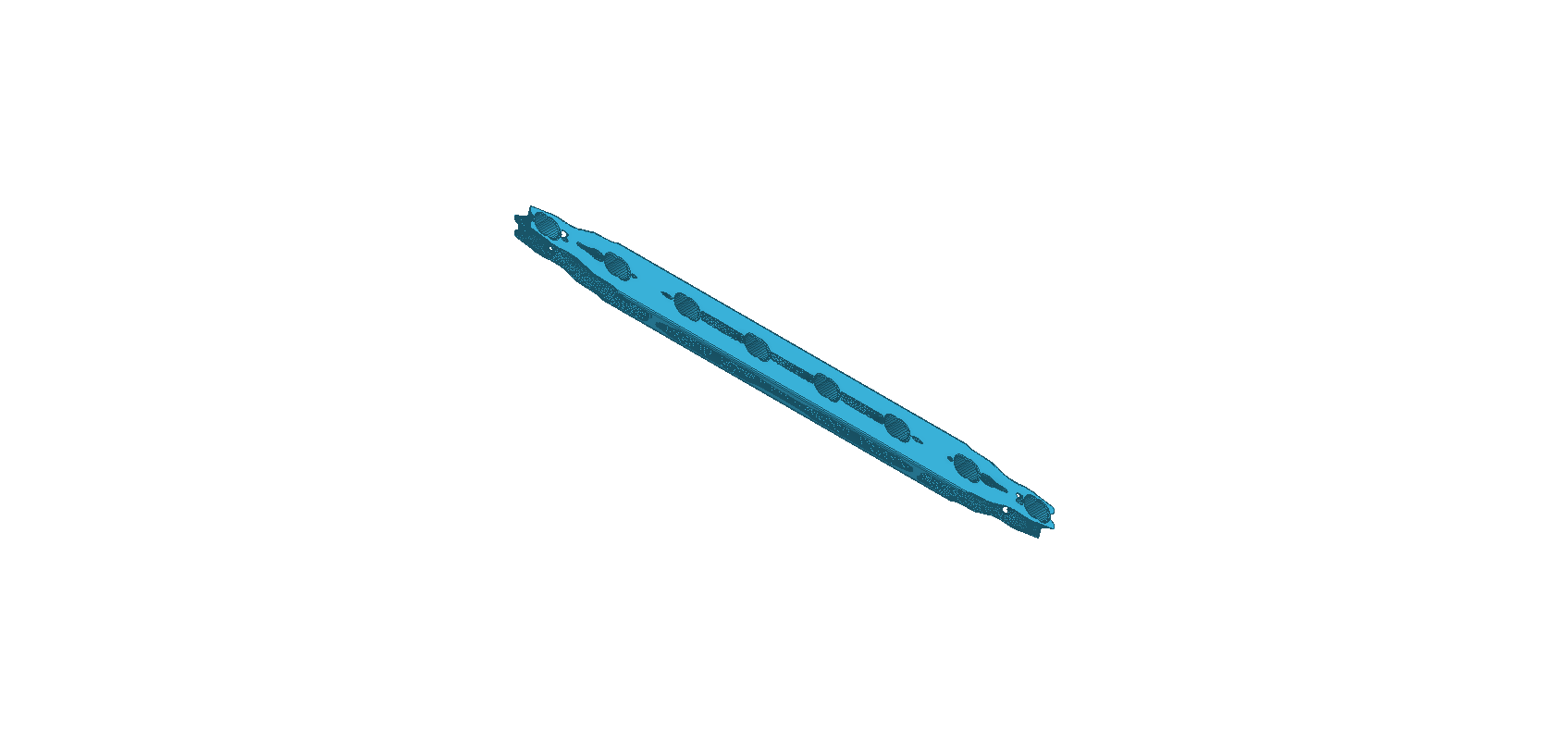  (c) |
| --- | --- | --- |
| 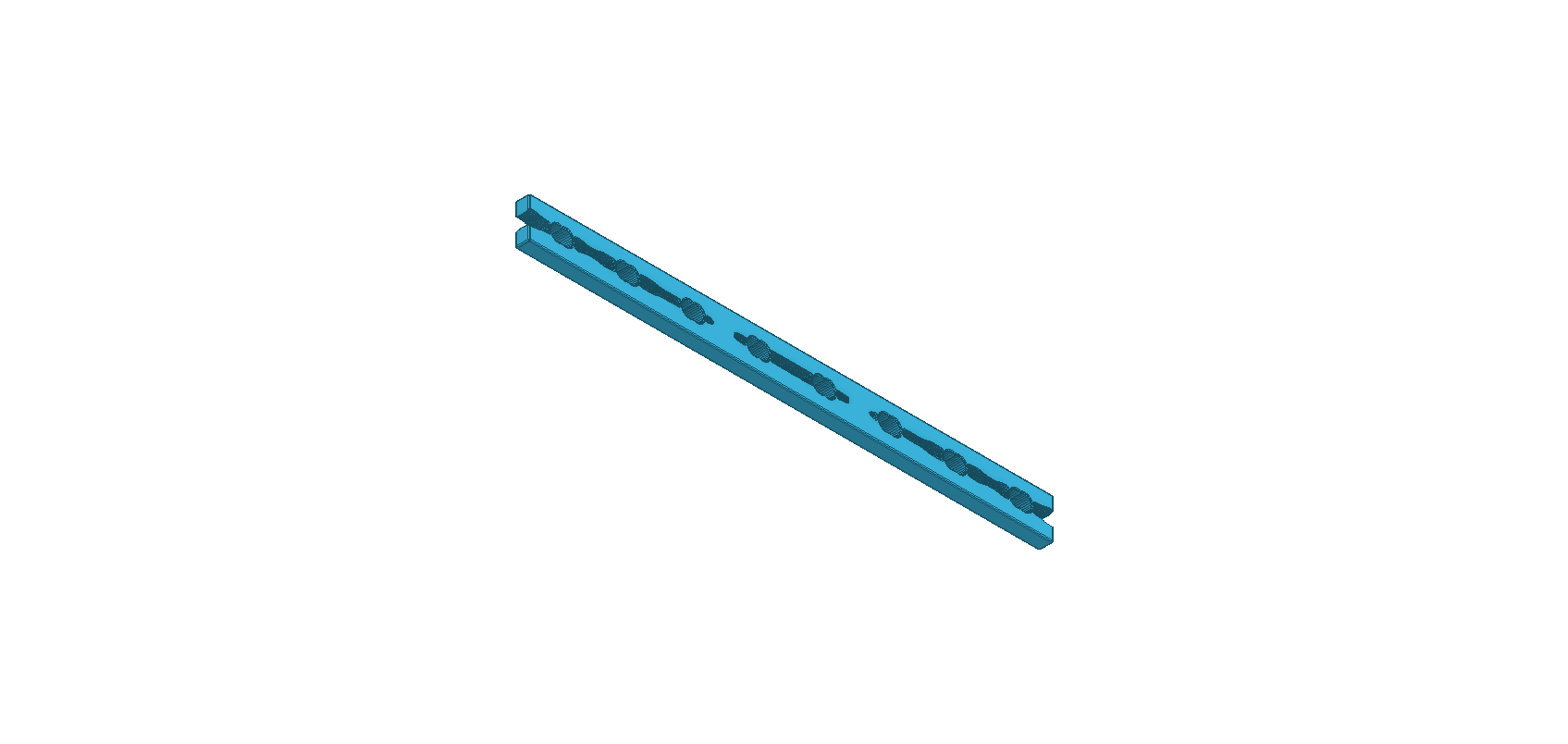  (d) | 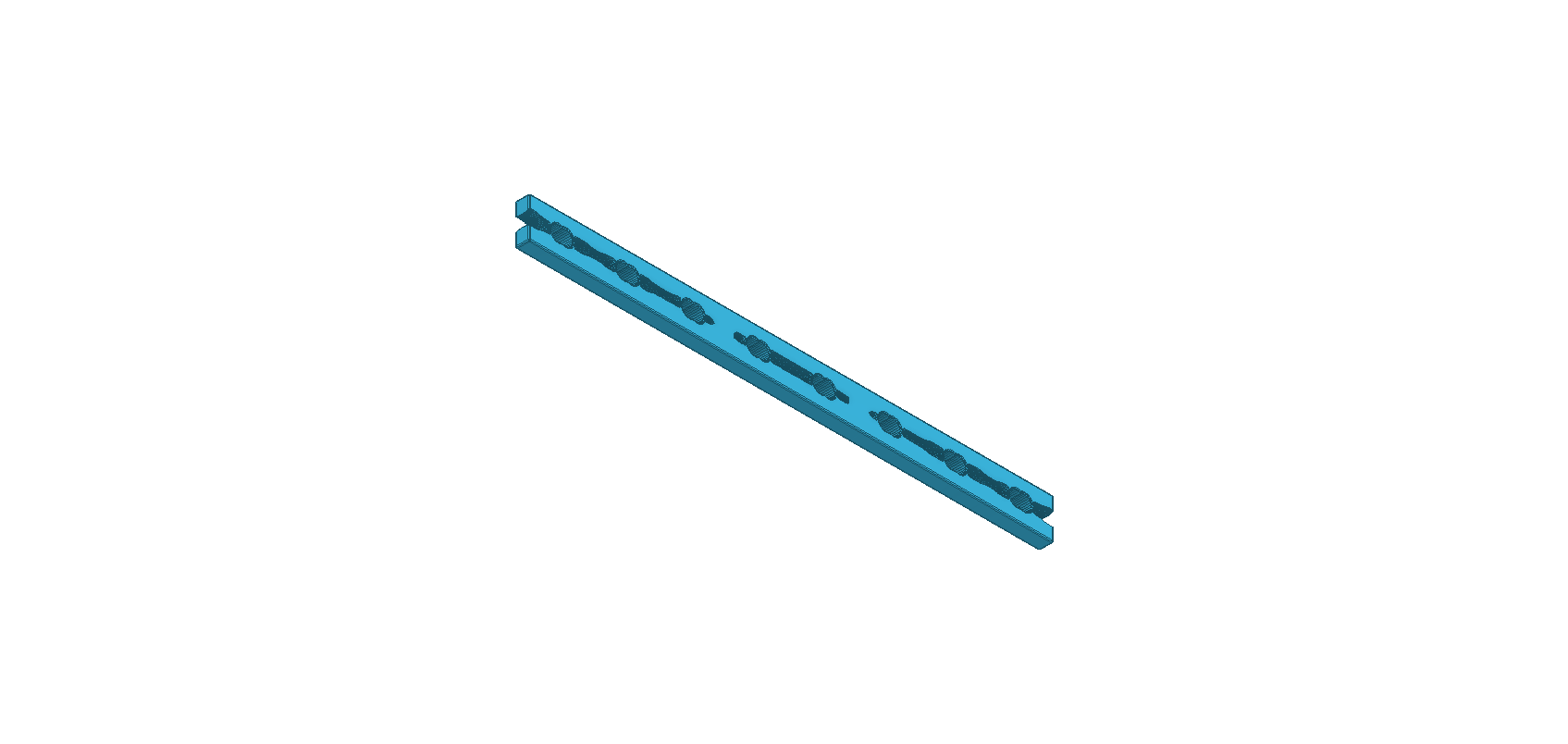  (e) | 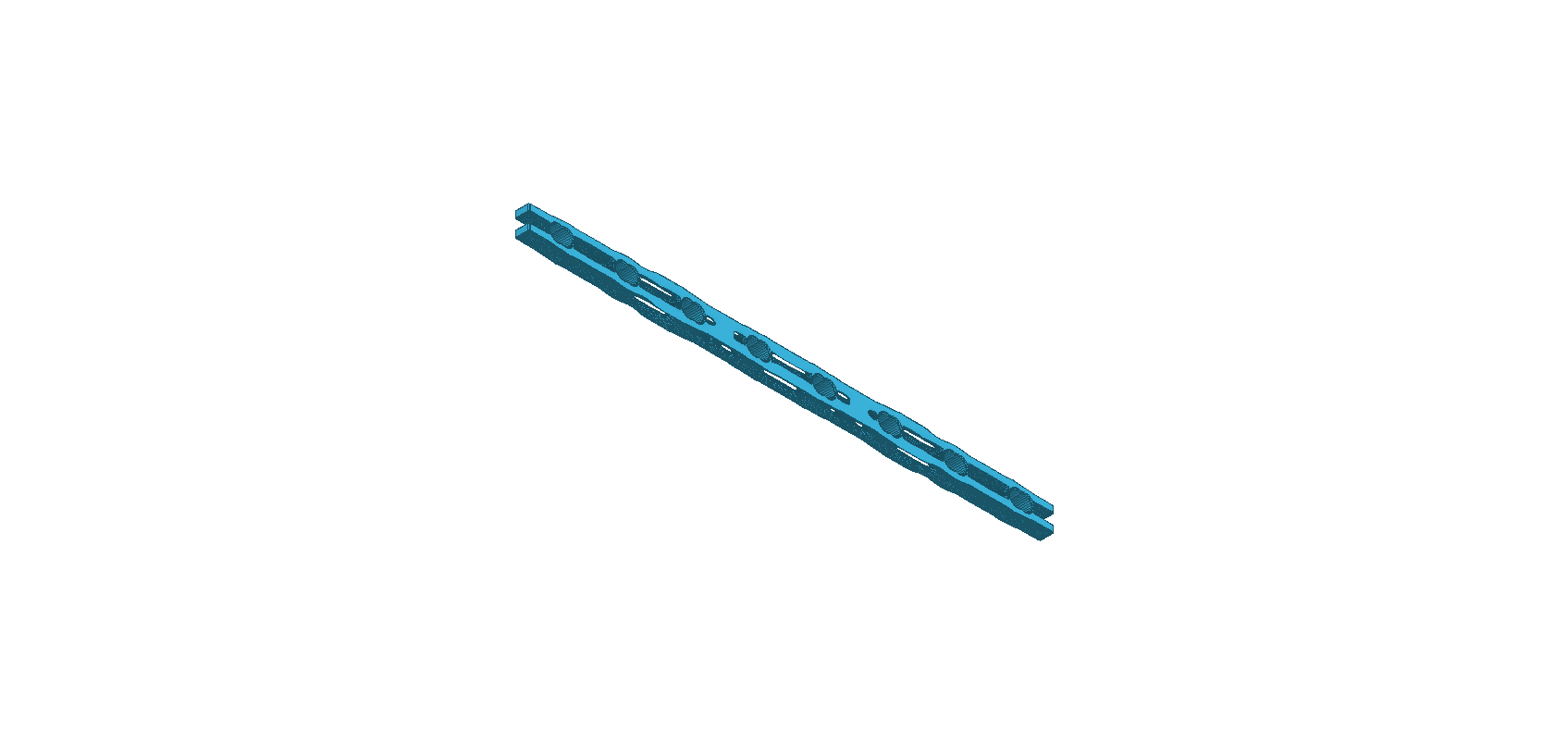  (f) |
| 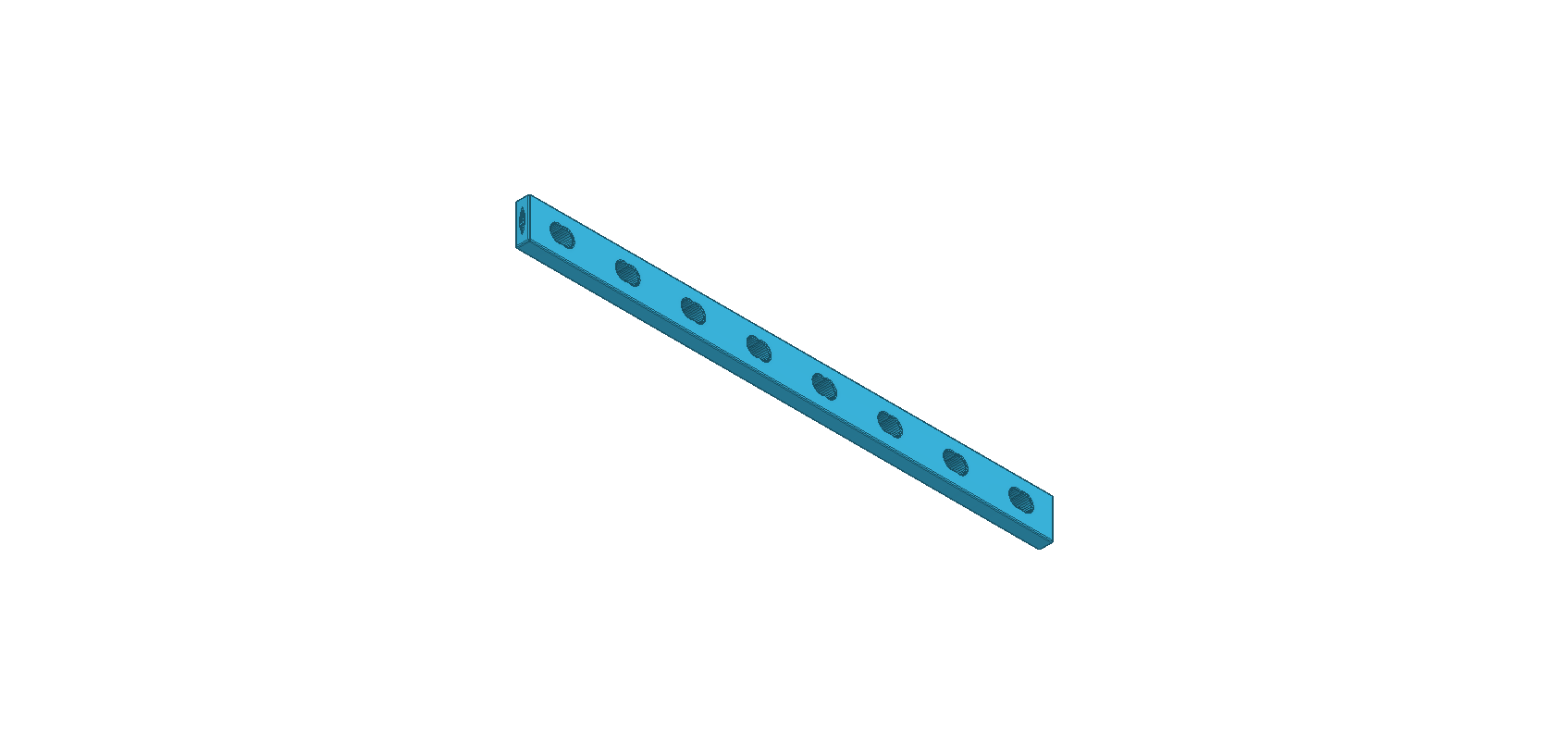  (g) | 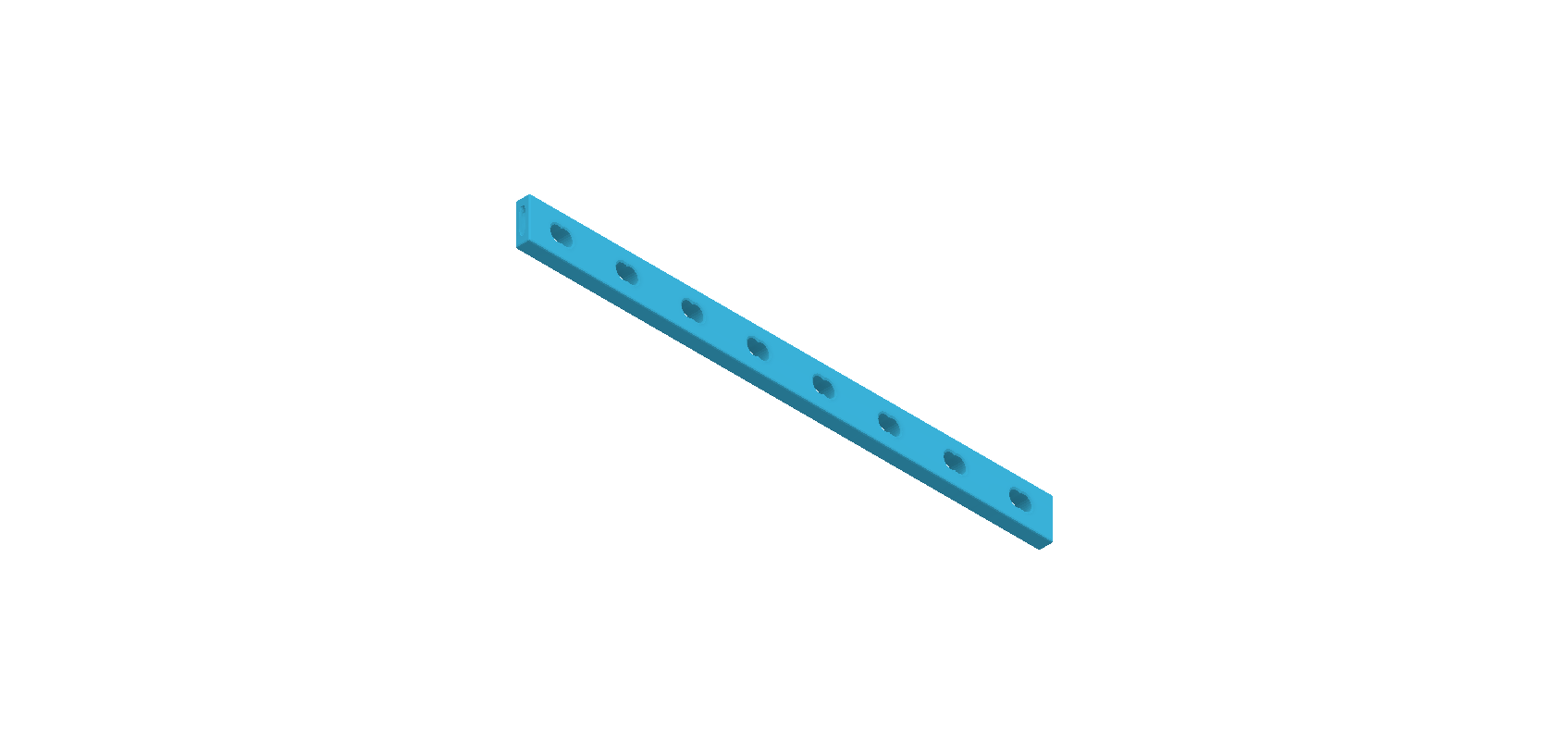  (h) | 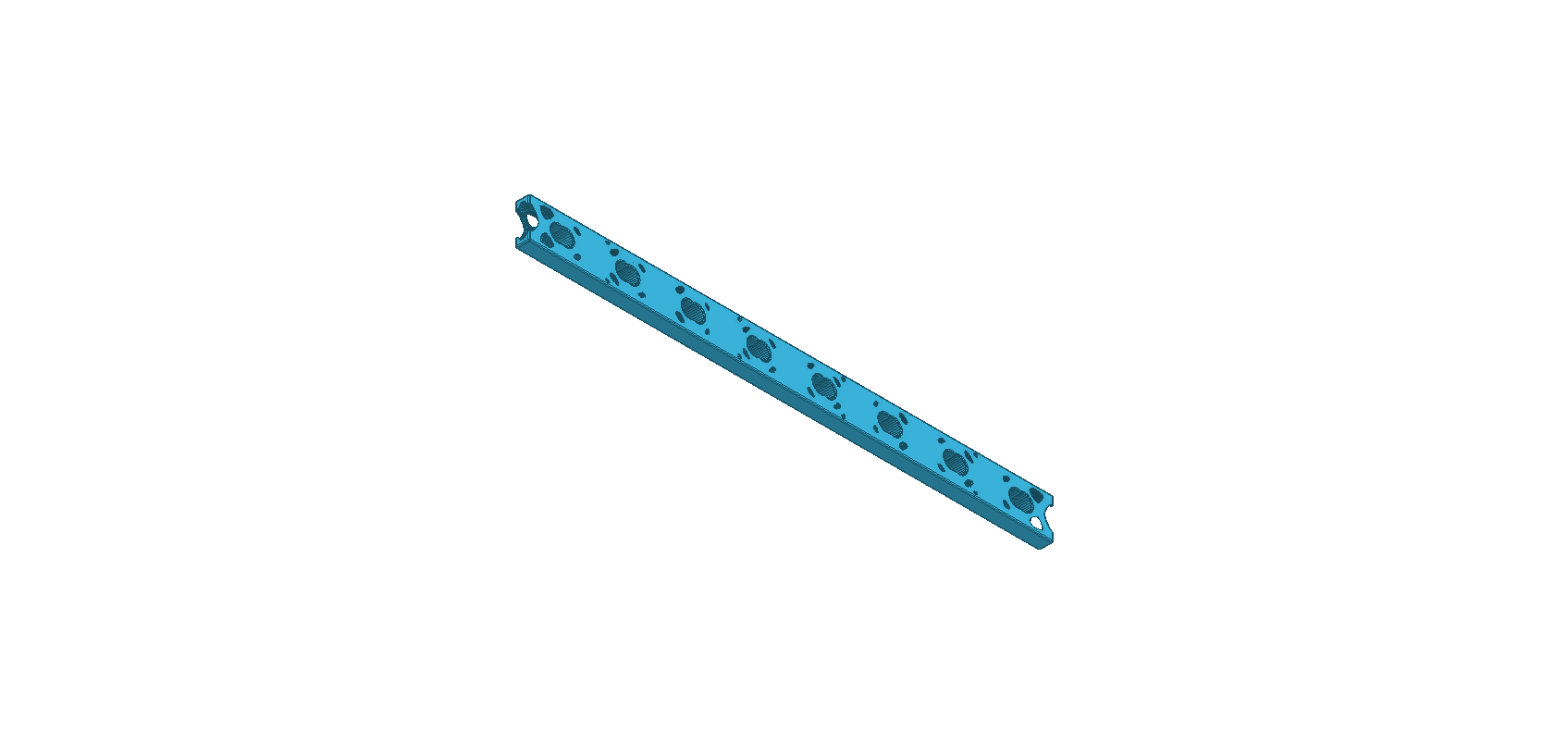  (i) |
| 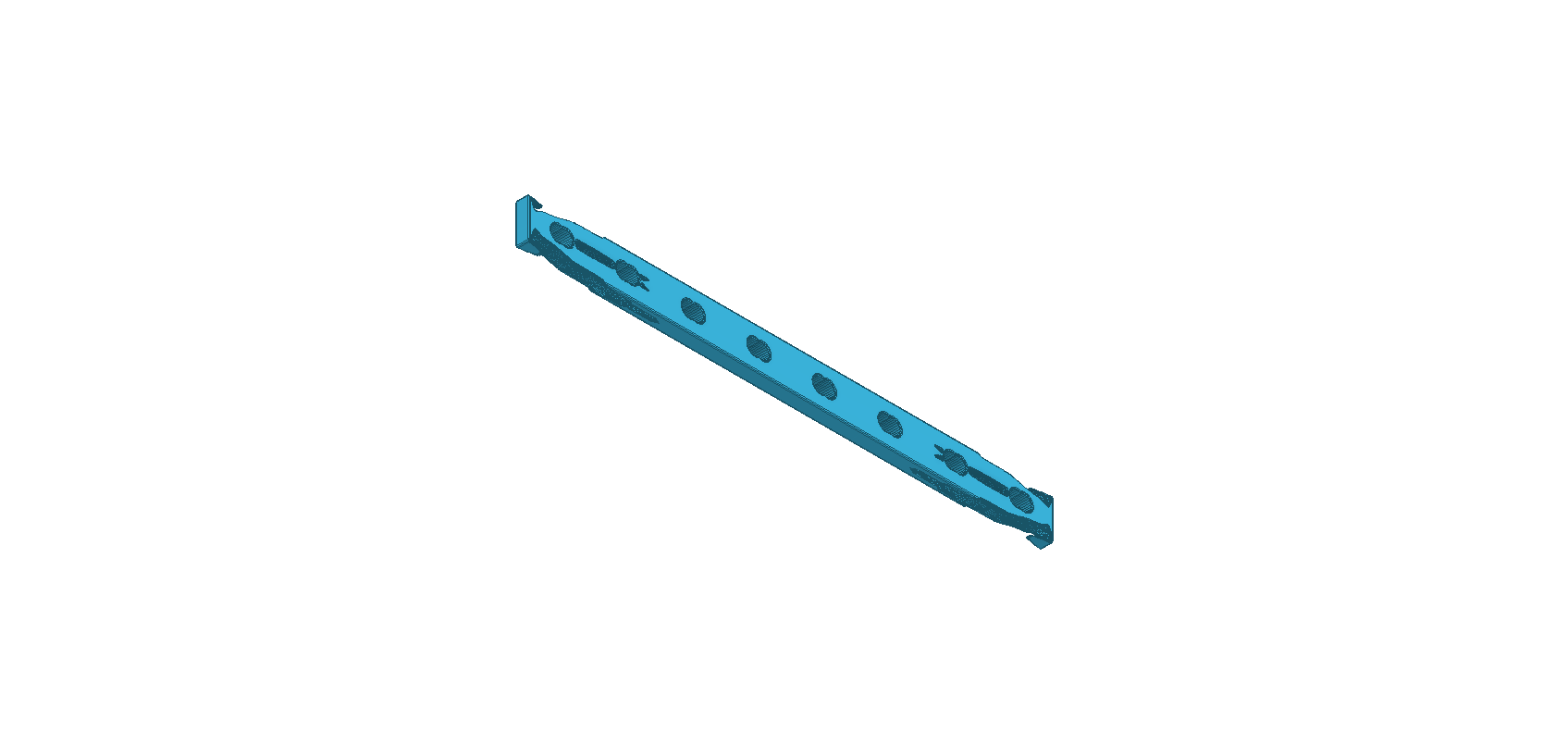  (j) | 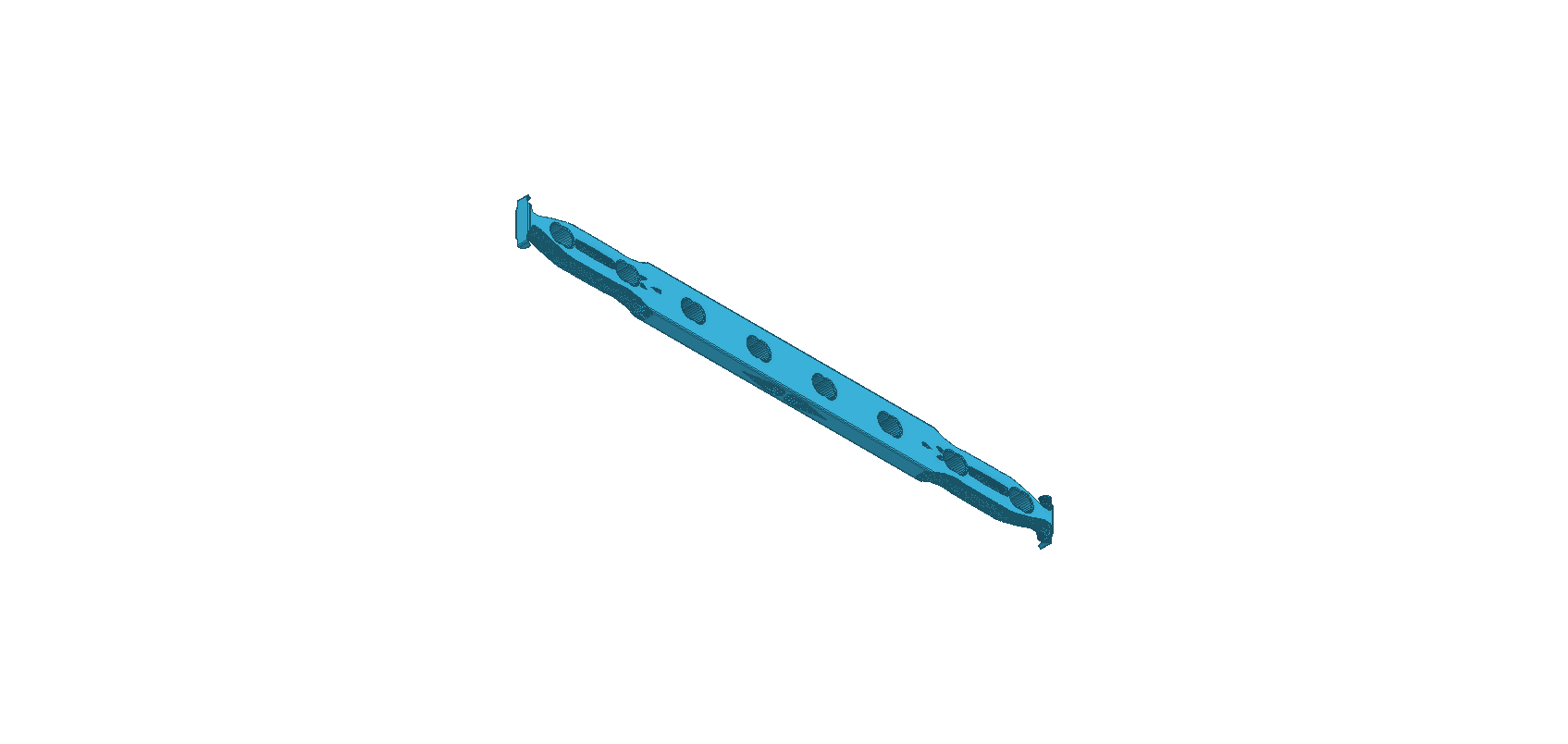  (k) | 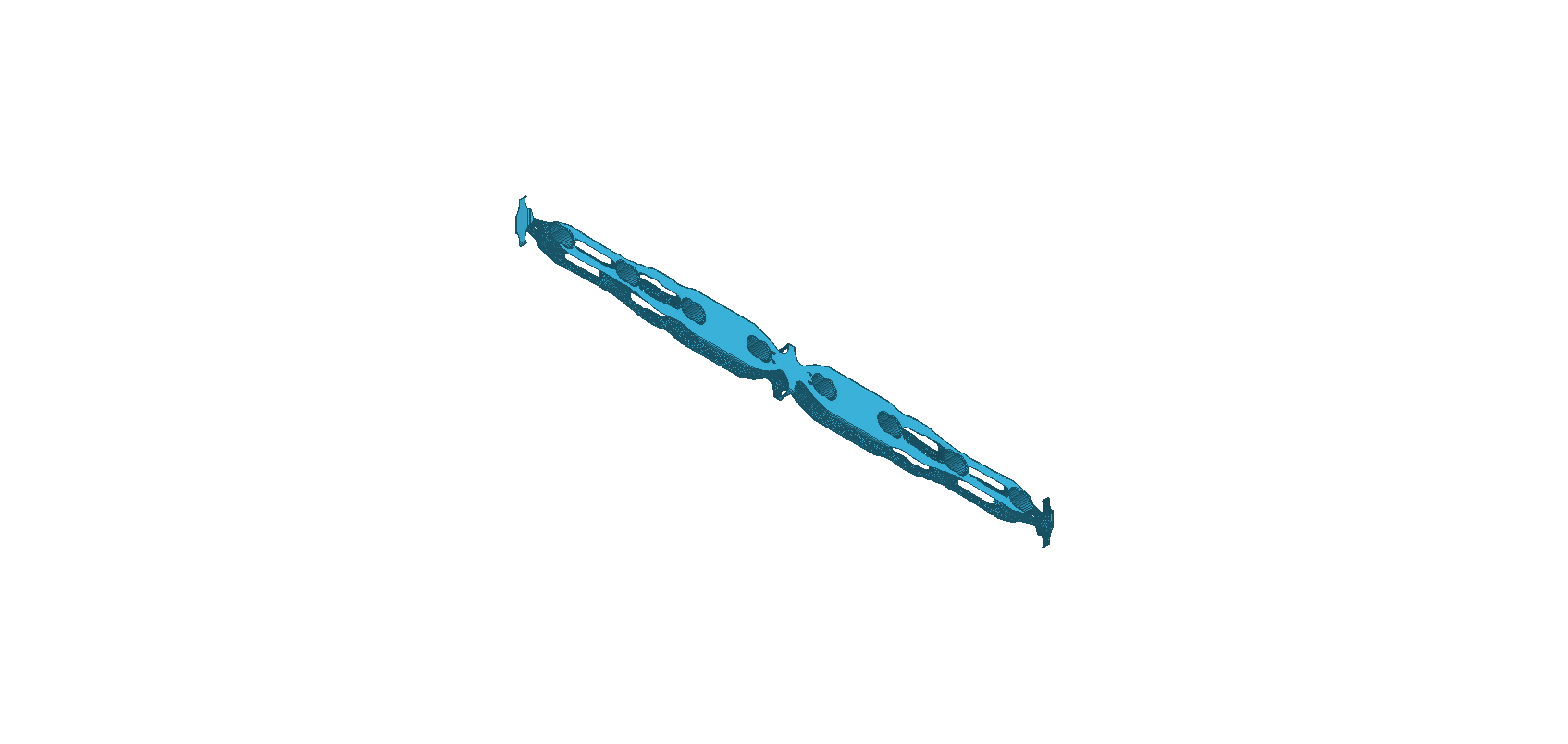  (l) |

Fig. S2 Topology optimization results of eight-hole fracture plate. Under bending load: (a) 25% of volume reduction, (b) 45% of volume reduction and (c) 75% of volume reduction. Under compression load: (d) 25% volume reduction, (e) 45% of volume reduction and (f) 75% volume reduction. Under torsion load: (g) 25% of volume reduction, (h) 45% of volume reduction and (i) 75% of volume reduction. Under combined load: (j) 25% of volume reduction, (k) 45% of volume reduction and (l) 75% of volume reduction.
